# Biomedical Application of Reactive Oxygen Species–Responsive Nanocarriers in Cancer, Inflammation, and Neurodegenerative Diseases

**DOI:** 10.3389/fchem.2020.00838

**Published:** 2020-09-18

**Authors:** Jinggong Liu, Yongjin Li, Song Chen, Yongpeng Lin, Haoqiang Lai, Bolai Chen, Tianfeng Chen

**Affiliations:** ^1^Orthopedics Department, Guangdong Provincial Hospital of Traditional Chinese Medicine, The Second Affiliated Hospital of Guangzhou University of Chinese Medicine, Guangzhou, China; ^2^Department of Chemistry, Jinan University, Guangzhou, China

**Keywords:** reactive oxygen species, nanocarriers, cancer, inflammation, neurodegenerative diseases

## Abstract

Numerous pathological conditions, including cancer, inflammatory diseases, and neurodegenerative diseases, are accompanied by overproduction of reactive oxygen species (ROS). This makes ROS vital flagging molecules in disease pathology. ROS-responsive drug delivery platforms have been developed. Nanotechnology has been broadly applied in the field of biomedicine leading to the progress of ROS-responsive nanoparticles. In this review, we focused on the production and physiological/pathophysiological impact of ROS. Particular emphasis is put on the mechanisms and effects of abnormal ROS levels on oxidative stress diseases, including cancer, inflammatory disease, and neurodegenerative diseases. Finally, we summarized the potential biomedical applications of ROS-responsive nanocarriers in these oxidative stress diseases. We provide insights that will help in the designing of new ROS-responsive nanocarriers for various applications.

## Introduction

Oxygen is necessary for aerobic respiration in living bodies. It is required in oxidative metabolism for the generation of adenosine triphosphate. However, partial reduction of molecular oxygen is mutagenic as it leads to the formation of reactive oxygen species (ROS) (Kaelin and Thompson, 2010). ROS constitutes a collective terminology referring to oxygen-derived free radicals and small molecules consisting of superoxide anion (${\text{O}}_{2}\cdot^{-}$), hydroxyl free radical (·OH), hydrogen peroxide (H_2_O_2_), hypochlorous acid (HOCl), singlet oxygen (^1^O_2_), and so on (Bayr, 2005; Giorgio et al., 2007; Trachootham et al., 2009; Dickinson and Chang, 2011; Gligorovski et al., 2015). ${\text{O}}_{2}\cdot^{-}$ is the primary ROS produced by metabolic processes. Activation of oxygen with an electron from physical irradiation produces ${\text{O}}_{2}\cdot^{-}$, which generates ROS through a series of reactions. ${\text{O}}_{2}\cdot^{-}$ directly interacts with other molecules through enzymatic or metal-catalyzed processes to produce secondary ROS (Imlay, 2003; Valko et al., 2006; Hayyan et al., 2016). ROS is indispensable for normal physiological functions as they participate in cell signaling, immunity, and tissue homeostasis (Bryan et al., 2012; Ray et al., 2012; Nathan and Cunningham-Bussel, 2013; Nosaka and Nosaka, 2017).

ROS is considered as a double-edged sword playing beneficial or unavoidable toxic functions in living systems, depending on the equilibrium between ROS production and antioxidants. ROS is essential for physiological metabolism at normal concentrations. They regulate cellular response to hypoxia and resistance to infectious agents and participate in several cell-signaling systems. However, very high or low ROS levels directly or indirectly result in the pathogenesis of various diseases (Bandyopadhyay et al., 1999; Di Rosanna and Salvatore, 2012; Franceschi et al., 2018). Generally, numerous substances in cells are susceptible to the effects of ROS. ROS causes cellular damage and results in the formation of harmful by-products, such as lipid oxides and lipid peroxides. Similarly, excessive ROS causes damage proteins and DNA, blocks enzyme activity, or even leads to cancer (Kumar et al., 2008; Mouthuy et al., 2016; Kunkemoeller and Kyriakides, 2017; Franceschi et al., 2018). The imbalance in ROS generation and elimination is believed to be implicated with the oxidative stress, resulting in mitochondrial dysfunction. Oxidative stress directly or indirectly causes various diseases (Andersen, 2004; Barnham et al., 2004; Houstis et al., 2006; Ishikawa et al., 2008; Fraisl et al., 2009; Trachootham et al., 2009), including stroke (Sarmah et al., 2019), sepsis (Hoetzenecker et al., 2012), diabetes (Liang et al., 2018), hypertension (Touyz, 2003), neurodegenerative diseases (Radi et al., 2014), inflammation (Blaser et al., 2016), and cancer (Schumacker, 2015). Restoring the appropriate ROS concentration by regulating ROS production or neutralizing ROS is a potentially effective means of preventing and treating diseases related to oxidative stress (Zhou et al., 2016).

The unique redox microenvironment distinguishes the pathological area from the surrounding normal environment (Forman and Torres, 2001; Gomberg, 2020). For instance, the concentration of H_2_O_2_ in healthy human plasma is ~1 to 8 μM (Lacy et al., 2000), whereas its level following activation of macrophages is as high as 1,000 μM (Droge, 2002; Yao et al., 2019). The concentration of hydrogen peroxide in respiratory lining cells is nearly 0.1 to 1 μM, but this increases by 20-fold in cases of inflammatory lung disease (Sznajder et al., 1989; Burgoyne et al., 2013). Developing ROS-responsive agents is postulated to be a promising solution to control the detrimental effects of ROS in cells (Liang and Liu, 2016; Hu et al., 2017; Zhang et al., 2019). Changes in the chemical structure, biochemical, or physical properties of ROS-responsive materials are induced by environmental stimuli (e.g., light, enzymes, pH, ionic strength, temperature, etc.) (Wang et al., 2014; Fang et al., 2015; An et al., 2016; Dou et al., 2017; Lee et al., 2018; Qiao et al., 2018; Xiang et al., 2018; Yang et al., 2020b). So far, stimuli-responsive agents have been extensively studied in biotechnology, as well as biomedicine fields (Hoffman, 2013; Grzelczak et al., 2019; Ovais et al., 2020). Redox-reactive materials hold huge promise in the design of drugs and gene delivery systems to target site-specific disease sites based on overproduction of ROS, protecting the cells against oxidative stress. This is because they can sense and eliminate active oxygen.

**Figure 1:**
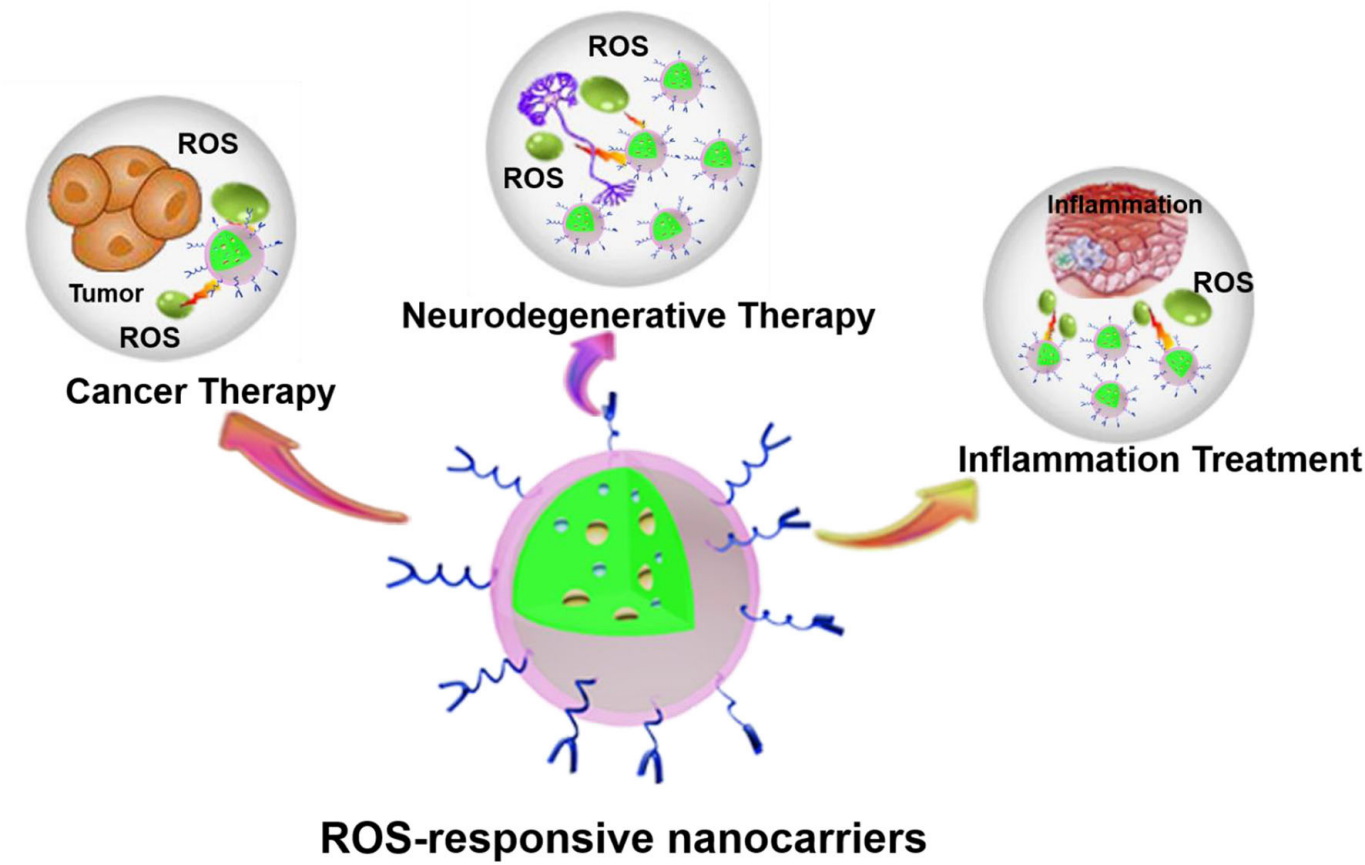
ROS-responsive nanocarriers for various applications. The abundant material chemistry endows nanocarriers with unique ROS-responsive properties for the treatment of various pathological diseases, such as cancer, inflammation, neurodegenerative diseases, etc.

Nanotechnology provides numerous applications in the field of biomedicine. The development of nanotechnology has resulted in considerable progress in the design of nanoparticles (NPs) targeting ROS responses. Many researchers have studied the preparation and application of some ROS-responsive NPs (Tapeinos and Pandit, 2016; Xu et al., 2016; Saravanakumar et al., 2017; Ballance et al., 2019; Fan and Xu, 2020). Herein, we focused first on the production and physiological/pathophysiological effects of normal levels of ROS. Then the roles of ROS in cancer, inflammatory diseases, and neurodegenerative diseases were clarified. Moreover, we also focused on the latest progress of various ROS-responsive nanocarriers and highlight the mechanisms by which nanocarriers respond to changes in the oxidative microenvironment and its potential biomedical applications in three aspects, including cancer, inflammatory diseases, and neurodegenerative diseases (Figure 1).

## ROS Production and Physiological/Pathophysiological Effects

### The Generation of ROS

**Figure 2:**
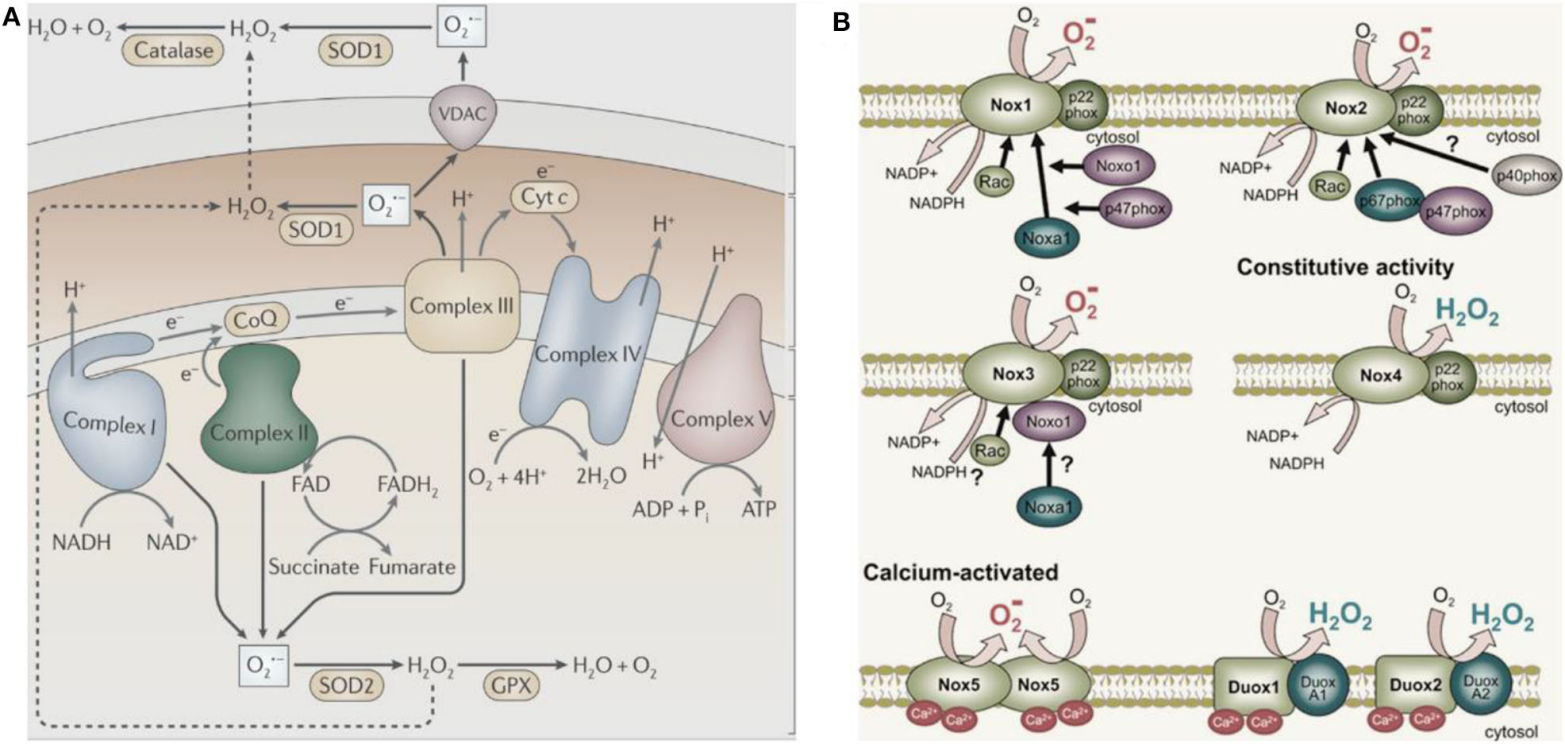
The major cellular sources of ROS production. **(A)** The mitochondria sources of ROS (West et al., 2011) (Copyright 2011, reproduced with permission from Elsevier). **(B)** The NADPH sources of ROS (Brandes et al., 2014) (Copyright 2014, reproduced with permission from Elsevier).

In 1954, Commoner et al. (1954) discovered free radicals in various freeze-dried biological materials. In the same year, Gerschman (1954) proposed that oxidized free radicals derived from partially reduced oxygen cause oxygen poisoning and related diseases. Harman (1956) subsequently defined these oxidizing free radicals and small molecules as ROS. ROS is produced by healthy cells during metabolism and in specific subcellular compartments (mainly mitochondria, Figure 2A, West et al., 2011). Activation of the nicotinamide adenine dinucleotide phosphate oxidase (NOX) complex located in the cell membrane generates ROS in some cancer cells (Figure 2B, Brandes et al., 2014). The endoplasmic reticulum also produces ROS; for example, flavoenzyme endoplasmic oxidoreductin-1 uses O_2_ as a 2-electron receptor to produce H_2_O_2_ (Nathan and Cunningham-Bussel, 2013). ${\text{O}}_{2}\cdot^{-}$, as the first generated ROS, acts as a signaling molecule in aerobic organisms and regulates multiple physiological processes, including cell aging, apoptosis, and host defense (Newsholme et al., 2016). The monovalent reduction between O_2_ and NADPH molecules generates ${\text{O}}_{2}\cdot^{-}$, which is converted to H_2_O_2_ and has a relatively short biological life span (Bhattacharjee, 2019). Notably, H_2_O_2_ is the most stable form of ROS and diffuses freely within and between cells. Besides direct oxidative injury, ${\text{O}}_{2}\cdot^{-}$and its by-product H_2_O_2_ participate in the formation of other reactive substances (Bolduc et al., 2019). For instance, ${\text{O}}_{2}\cdot^{-}$ results in the production of highly reactive hydroxyl groups (·OH) through the Haber-Weiss cycle or a Fenton-type reaction.

HOCl is another type of ROS produced by the catalytic reaction of myeloperoxidase (MPO) and eosinophil peroxidase with H_2_O_2_ and Cl^−^ in body fluids (Freitas et al., 2009). HOCl is 100 to 1,000 times more destructive than ${\text{O}}_{2}\cdot^{-}$ and H_2_O_2_. Moreover, when it reacts with other biologically active molecules, it produces even more toxic effects than O^2·−^ and H_2_O_2_. HOCl promotes the production of ^1^O_2_, a free radical with high reactivity with many biomolecules (Winterbourn, 2008). ·OH, HOCl, and ^1^O_2_ are considered secondary ROS in comparison with the original ROS (${\text{O}}_{2}\cdot^{-}$ and H_2_O_2_). They cause higher oxidative damage to cells and tissues. Therefore, under stress conditions, it is crucial to prevent secondary ROS-induced oxidative damage. Unfortunately, there are no endogenous protective enzymes specific for these secondary ROS.

### The Effect of Normal Levels of ROS on Physiological/Pathophysiological Process

A basal level of ROS can participate in many important physiological processes and play an important role in various signal cascades, such as the responses to growth factor stimulation or inflammation (Finkel, 2011; Le Belle et al., 2011; Beckhauser et al., 2016). ROS is involved in numerous cellular processes, including cell growth, proliferation, differentiation, apoptosis, cytoskeleton regulation, contraction, and migration. A healthy human body needs protection from the inflammatory responses, which eliminate harmful stimuli and initiate the healing process (Clark, 1999; Lamkanfi and Dixit, 2012). The NOX complex is rapidly activated by soluble factors and stimulants through interactions with cell surface receptors, leading to massive ROS oxidation (Lambeth, 2004). Numerous studies have confirmed that ROS acts as secondary messengers to regulate the production of inflammatory molecules and cytokines (Blaser et al., 2016; Missiroli et al., 2020). For instance, ROS stimulates macrophages to release tumor necrosis factor and the proinflammatory cytokine interleukin 1 (IL-1) (Hsu and Wen, 2002).

Active cellular proliferation produces H_2_O_2_, which influences the proliferation and differentiation of stem cells. For example, H_2_O_2_ is produced during proliferation of adult neural hippocampal progenitor cells, which then regulate self-renewal as well as neurogenesis via the PI3K/Akt signaling cascade. H_2_O_2_ augments overall proliferation of neural stem cells (NSCs) at moderate concentrations (Le Belle et al., 2011). At the same time, H_2_O_2_ is also considered as an intracellular signaling medium for neuronal differentiation induced by the nerve growth factor (Suzukawa et al., 2000). The rare case of hypothyroidism has revealed another example of the importance of ROS in health (Erdamar et al., 2008). H_2_O_2_ is an essential cofactor for thyroid peroxidase, which is involved in the production of thyroid hormone. Numerous studies have shown that dual oxidase 2 (and possibly dual oxidase 1) enzyme is required for H_2_O_2_ production and thyroid peroxidase function (Ameziane-El-Hassani et al., 2005). Notably, patients with congenital hypothyroidism possess dual oxidase 2 gene mutations, providing strong support for this theory (Moreno et al., 2002).

## Mechanisms and Effects of Abnormal ROS Levels on Oxidative Stress Diseases

### Cancer

Globally, cancer, following the cardiovascular disease, has the second highest death rate. Chemotherapy is currently among the primary clinical therapies for cancer; however, it is well-known to have relatively serious side effects. While chemotherapy drugs kill tumor cells, they additionally kill healthy cells and severely damage the immune system (Blattman and Greenberg, 2004). At the same time, multiple rounds of treatment with chemotherapeutic drugs cause cancer cells to become resistant, rendering the chemotherapy drugs ineffective (Riley et al., 2019). ROS has a critical influence on the progression of the cell cycle of malignancy cells through their role in energy metabolism, cell movement, cell state maintenance, cell proliferation, and apoptosis (Liou and Storz, 2010). Notably, ROS plays a dual function in tumors; they promote tumor proliferation, survival, and adaptation to hypoxia (Tafani et al., 2016). Cancer cells increase their metabolism and adapt to hypoxia to increase their ROS production rate to overactivate cancer-promoting signaling. On the other hand, ROS promotes antitumor signaling and triggers cancer cell death induced by oxidative stress (Reczek and Chandel, 2017). Moreover, ROS promotes tumorigenic signal transduction by overactivating the PI3K/Akt/mTOR survival cascade and by oxidation and deactivation of phosphatase and tensin homolog deleted on chromosome ten (PTEN) and protein tyrosine phosphatase 1B (PTP1B) phosphatase (negative modulators of PI3K/Akt signal transduction). The carcinogenic stimulation of Akt elevates ROS production to additionally promote cancer cell proliferation and survival (Clerkin et al., 2008; Cairns et al., 2011). Other relationships between cancer and ROS have also been elucidated. For example, ROS promote tumor cell survival by activating nuclear factor κB and Nrf2 (transcription factors that up-modulate antioxidant expression), which enable malignancy cells to escape ROS-mediated cell apoptosis (Morgan and Liu, 2011). ROS production decreases with the destruction of the mitochondrial respiratory chain, reducing the occurrence of tumors (Weinberg et al., 2010). In response to glucose and hypoxia deficiency, cancer cells undergo metabolic transformations, including AMP protein kinase activation, hypoxia-inducible factor (HIF) stabilization, and the use of a carbon metabolism axis (Denko, 2008). This raises the production of NADPH and ROS while leading to tumor angiogenesis and metastasis (Ye et al., 2014). ROS formation is additionally promoted by the release of ${\text{O}}_{2}\cdot^{-}$, ·OH, and H_2_O_2_ from the mitochondrial electron transport chain. ROS then stabilizes HIF-1α in normoxia and hypoxia (Huang et al., 1996). ROS plays an essential role in cancer cell metastasis by stimulating matrix metalloproteinases that break down constituents of the extracellular matrix to promote cancer cell invasion and infiltration (Folgueras et al., 2004). This stimulates the formation of the infiltrating foot, a membrane protrusion in cancer cells that is rich in actin, and participates in the proteolysis and invasion behavior of malignancy cells (Morry et al., 2017).

### Inflammatory Diseases

Inflammation is related to many types of diseases, such as arthritis, coronary heart disease, myocardial infarction, asthma, and cystic fibrosis (Franceschi et al., 2018). Mounting research evidence shows that the overproduction of free radicals at the inflammatory area is related to the pathogenicity of associated diseases (Droge, 2002) Notably, ROS production has been reported to stimulate NLRP3 inflammatory body assembly in a ROS-sensitive manner (Hughes and O'Neill, 2018). The primary source of ROS in response to harmful stimuli constitutes the mitochondria, which also directs inflammation by releasing mitochondrial DNA. Uncontrolled ROS production by mitochondria, hyperactivated leukocytes, and endothelial cells under chronic inflammation eventually leads to serious cell and tissue damage, further promoting and aggravating inflammatory damage. In numerous inflammatory diseases, the presently available intervention approaches have limited or no success (Hotamisligil, 2017); hence, we require new methods of treating chronic inflammatory diseases. It is assumed that the persistence of oxidative stress promotes these harmful inflammatory processes and could serve as new targets for treating chronic inflammation (Mittal et al., 2014). Rheumatoid arthritis (RA) is a systemic autoimmune disease with unknown etiology typified by chronic joint pain, macrophage invasion, and activated T-cell infiltration. The redox-sensitive signaling cascades cause abnormal expression of several adhesion molecules related to RA, which also cause monocytes and lymphocytes to migrate into the synovium in RA patients (Hitchon and El-Gabalawy, 2004). Atherosclerosis is a disease characterized by arterial wall thickening and is considered an inflammatory disease because it promotes the recruitment, expansion, and maintenance of monocytes/macrophages. This is due to the expression of endothelial cell factors constituting adhesion molecules and chemoattractants (Kinscherf et al., 1999) Oxidative stress induces overexpression of protein kinases and intercellular adhesion molecules, further promoting the infiltration of monocytes, smooth muscle cells, and macrophages (Droge, 2002). These cells bind to oxidized low-density lipoprotein, activate monocytes as well as macrophages, stimulate the Mn superoxide dismutase expression, and increase the levels of H_2_O_2_ (Yang et al., 2017). This high aggregation of ROS is thought to contribute to the development of atherosclerosis by causing high levels of macrophage apoptosis (Kinscherf et al., 1999).

### Neurodegenerative Diseases

Diseases in which neuronal loss progresses slowly are collectively referred to as neurodegenerative diseases (Manoharan et al., 2016). The common neurodegenerative diseases include amyotrophic lateral sclerosis (ALS), Parkinson disease (PD), Huntington disease (HD), and Alzheimer disease (AD). The brain has a high requirement for oxygen and a comparatively high level of redox-active metals, e.g., iron or copper, which play catalytic roles in the production of ROS (Cheignon et al., 2018). Consequently, the brain is more susceptible to suffer from oxidative stress (Liu et al., 2017c). Additionally, as the concentration of polyunsaturated fatty acids in the cell membrane increases, the brain becomes more prone to lipid peroxidation (Youdim et al., 2000; Barnham et al., 2004). The causes of neurodegenerative diseases are closely related to oxidative stress (Uttara et al., 2009; Melo et al., 2011). Analysis of AD pathogenesis revealed that the deterioration of antioxidant status, mitochondrial deterioration, and increased apoptosis accompany poor antioxidant status (Manoharan et al., 2016). Physiologically, the brain has a low antioxidant capacity, and the glial cells and neurons have a relatively strong metabolism and higher oxidation sensitivity and are more likely to produce excessive superoxide free radicals, altogether making the brain more prone to oxidative stress, which causes AD (Nakajima and Kohsaka, 2001; Gadoth and Göbel, 2011). Similarly, oxidative stress is closely related to the pathogenesis of PD, ALS, and HD. The occurrence of diseases, such as PD, also leads to excessive ROS production. Numerous studies have shown that PD reduces the activity of the respiratory chain complex I, resulting in excessive ROS (Schapira, 1998; Guo et al., 2013). At the same time, dopamine metabolism at the site of the disease increases, causing the accumulation of toxic oxidative free radicals (Cadet and Brannock, 1998).

### ROS-Responsive Nanocarriers and Their Applications

The results of our group and numerous other studies show that ROS could be used as a target or biosignature for the treatment of various diseases (Huang et al., 2017, 2020; Mei et al., 2018; Lai et al., 2019; He et al., 2020; Yang et al., 2020a,c; Zhao et al., 2020). Reactive oxygen species–responsive materials refers to materials capable of responding to those elevated ROS, such as H_2_O_2_, O^2·−^, ^1^O_2_, and so on. There have been great progress in nanomedicines and responsive materials used in biomedical fields (Tao et al., 2017, 2019; Qiu et al., 2018, 2019; Luo et al., 2019; Feng et al., 2020; Hu et al., 2020; Kong et al., 2020; Tang et al., 2020; Xie et al., 2020). ROS-responsive nanocarriers have some unique advantages for therapy compared with these reported materials. ROS-reactive agents are activated by ROS *in vivo* to produce corresponding physical or chemical changes. ROS-reactive materials could be utilized as imaging agents, site-specific delivery agents, and drugs for the treatment of various diseases. They could additionally be employed to modulate the tissue microenvironment and enhance the regeneration of tissues, as well as navigating and sensing via programmed changes in material properties (Tapeinos and Pandit, 2016; Saravanakumar et al., 2017; Ballance et al., 2019). Depending on its reaction to oxidation, the mechanisms of ROS-reactive functional groups are categorized into two main classes, namely, the change of physical characteristics (solubility) and the change in chemical bonds accompanying polymer degradation. The responsiveness of these ROS-responsive functional groups under diverse conditions is dependent on the type of ROS, the structure of the polymer, the shape of the material, and the exposure time.

So far, top-down and bottom-up are the two main methods for the fabrication of NPs, including the nanocarriers (Chan and Kwok, 2011; Qiu et al., 2018). Generally, most of the ROS-responsive nanocarriers are formed by bottom-up methods. The small drug molecules or polymers can be built up into NPs with bottom-up methods, but the shape, size, and dispersity are not easily to control, while the top-down method can be used in the fabrication of NPs with well-controlled shape and uniform size (Chan and Kwok, 2011). Maruf et al. (2020) fabricated red blood cell membrane–coated ROS-responsive 5-aminolevulinic acid prodrug nanostructures with robust atheroprotection using the top-down method.

Drug carriers, in the form of NPs, which respond to ROS, are designed to release their payloads in response to high ROS levels by increasing emissions or explosions. The drug molecules contained in these particles are small compounds or biomolecules. The particles can be used with a group of chemicals that react with ROS to create a charge and change the hydrophilicity, bonding, breaking, or otherwise stimulate the reaction at the particle. Overall, these reactions cause the swelling of particles, separation of particles, or increased release of drug molecules from particles. Researchers choose specific mechanisms for particle operations and drug delivery, respectively, depending on the final target of the particles. Currently, most of the ROS-responsive nanocarriers reported show low toxicity toward their own *in vitro* and *in vivo* cell and animal evaluation models, and the loading capacity of these nanocarriers depends on the carrier itself and the interaction of between the carriers and the payloads. In this section, we will discuss NPs that respond to ROS and their use as drug delivery carriers for various applications in cancer, inflammation, and neurodegenerative diseases. Some representative ROS-responsive agents and their biomedical applications are also summarized in Table 1.

**Table 1:** Representative ROS-responsive materials and their biomedical applications.

**ROS-responsive materials**	**Nanocarriers**	**Application**	**References**
Selenium	Diselenide block copolymers	Oxidants and reductants dual-responsive combining radiotherapy and chemotherapy.	(Ma et al., 2010b)
	Diselenide-containing polyelectrolyte multilayer film	Combination of chemotherapy and photodynamic therapy	(Ren et al., 2013)
	Phosphate segments and selenide groups polymer	H_2_O_2_-triggered drug release for cancer treatment	(Liu et al., 2013)
	Selenium-containing polyphosphoester nanogels	ROS induced the release of Dox for cancer treatment	(Zhang et al., 2018)
	Selenium-containing amphiphilic block copolymer PEG-PUSe-PEG	Oxidation-responsive release of Dox	(Ma et al., 2010a)
Sulfur	Polypropylene sulfide nanoparticles	Reduce the tissue reaction to neuroprostheses	(Mercanzini et al., 2010)
	Free-blockage mesoporous Nanoparticles	ROS induced the release of Dox for cancer treatment	(Cheng et al., 2017)
	Thioether linked conjugates	GSH and ROS dual-responsive for cancer chemotherapy	(Luo et al., 2016)
	Thioketal nanoparticles (TKNs) loaded with TNF-α-siRNA	ROS-sensitive nanoparticles targeting inflammation with oral administration	(Wilson et al., 2010)
Tellurium	Coassemblies of tellurium-containing molecules and phospholipids	ROS-responsive with good biocompatibility	(Wang et al., 2015)
	Hyperbranched tellurium-containing polymers	Site-specific elimination of excess ROS	(Fang et al., 2015)
	Tellurium-containing polymer (PEG-PUTe-PEG) based nanoparticles	Near-infrared light stimuli-responsive synergistic therapy for cancer	(Li et al., 2017a)
	Tellurium-containing polymer micelle	Combination of chemo- and radio-therapies with responsive to both H_2_O_2_and 2 Gy gamma radiation	(Cao et al., 2015)
Oxalate esters	Poly(vanillin oxalate) (PVO) nanoparticles	H_2_O_2_-responsive nanoparticles for the treatment of ischemia–reperfusion injury	(Kang et al., 2016)
	Poly(vanillyl alcohol-co-oxalate) (PVAX) polymers	Oxidation-responsive nanoparticles for anticancer drug delivery	(Huang et al., 2018)
	Peroxalate nanoparticles	*In vivo* imaging of H_2_O_2_	(Lee et al., 2007)
	Hydroxybenzyl alcohol (HBA)-incorporated copolyoxalate	H_2_O_2_ responsive nanoparticles for detection and therapy of ischemia–reperfusion injury	(Lee et al., 2013)
	Poly(vanillin oxalate) (PVO)	H_2_O_2_- and acid-mediated hydrolytic degradation with anti-inflammatory activity	(Kwon et al., 2013)
Phenylboronic acid (ester)	Amphiphilic block copolymers containing aryl boronate ester-capped block	Sustained drug release combination chemotherapy with magnetic resonance (MR) imaging	(Deng et al., 2016)
	Conjugating phenylboronic acid pinacol ester (PBAP) groups onto β-CD	ROS-responsive and H_2_O_2_-eliminating materials for diseases associated with inflammation and oxidative stress	(Zhang et al., 2017)
	Boronic ester modified dextran polymer nanoparticles	H_2_O_2_ responsive nanoparticles for ischemic stroke treatment	(Lv et al., 2018)
	Poly[(2-acryloyl)ethyl(*p*-boronic acid benzyl)diethylammonium bromide] (BA-PDEAEA, BAP) modified traceable nanoparticles	H_2_O_2_ responsive nanoparticles for RNAi-based immunochemotherapy of intracranial glioblastoma	(Qiao et al., 2018)

### Application of ROS-Responsive NPs for Cancer Therapy

In cancer cells, the concentration of ROS is higher compared with the normal cells because of the constant generation of ROS as the by-products of aerobic metabolic processes resulting from oncogenic transformation (Kong and Chandel, 2020). The elevated ROS levels in malignancies are employed in the design of ROS-responsive nanoagents, which promote site-specific drug release. The most common typical groups utilized in the design of ROS-responsive components include boronic ester, thioketal, and sulfide groups (summarized in Table 1). These ROS-responsive components result in the design of drug carriers for the systematic delivery of chemotherapy.

**Figure 3:**
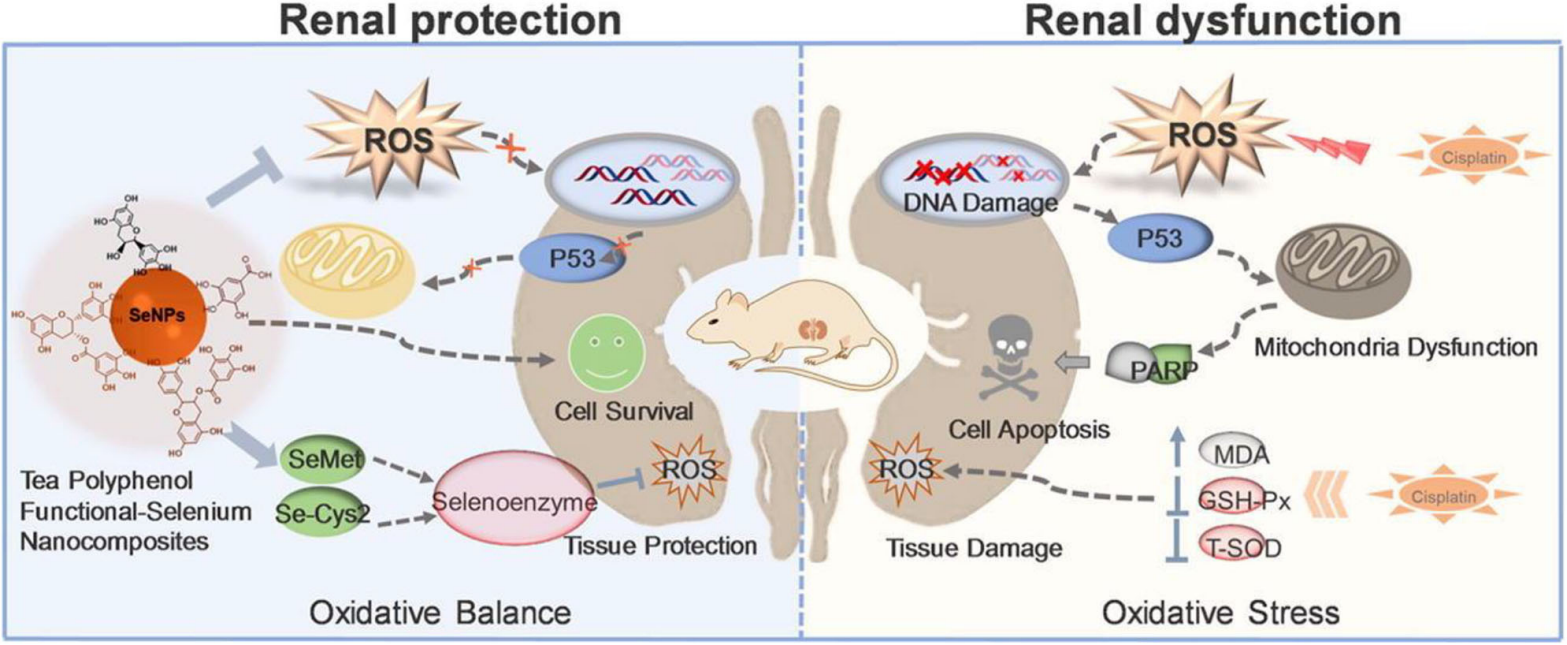
Tea polyphenol functionalized selenium nanoparticles reversed cisplatin-induced renal injury (Lai et al., 2019) (Copyright 2019, reproduced with permission from Elsevier).

Selenium (Se) is a chalcogen element widely present in some proteins capable of maintaining the cellular redox homeostasis (e.g., glutathione peroxidase, thioredoxin reductase) (Chaudhary et al., 2016). Se-containing particles can oxidize and change their hydrophilicity in response to ROS. Various Se derivatives of inorganic, organic, and amino acids have been found to exhibit biological activity primarily via antioxidant and pro-oxidant mechanisms (Lai et al., 2019; Huang et al., 2020). The different oxidation states (−2, 0, +4, +6) and forms of Se contribute to the antioxidant effects of Se. The direct antioxidant function of Se is conferred by some of the selenoproteins that directly protect against oxidative stress. Moreover, the regeneration of low-molecular-weight antioxidants (Q10, vitamins C and E, etc.) mediated by selenoproteins makes Se an indirect antioxidant (Hatfield et al., 2009; Lobanov et al., 2009). However, at elevated doses, Se typically turns into a pro-oxidant with well-established growth inhibiting properties and high cytotoxic activities. Toxicity of Se compounds is thus strictly dependent on the concentration of Se-binding chemical species and the associated redox potential (Weekley and Harris, 2013). Therefore, in addition to redox function of modified Se NMs, anticancer activity of differently modified Se NMs has also been reported (Liu et al., 2015, 2016). Epidemiological studies have shown that SeNPs can effectively prevent and treat diseases related to oxidative stress. The overproduction of ROS is an important contributor for cisplatin-induced nephrotoxicity. Our group developed a polyphenol-functionalized SeNPs (Se@TE NPs) using microwave-assisted method (Lai et al., 2019). Se@TE NPs showed renal protection activities through reducing the cisplatin-induced ROS. The facial tea polyphenols are ROS-responsive and could be consumed with the explosion to ROS, releasing the inner SeNPs. The suppressing of p53 phosphorylation and regulating of AKT and MAPKs signaling pathways of Se@TE NPs were confirmed in HK-2 cells. Further mechanistic studies suggested that Se@TE NPs showed its protective effects in the form of selenomethionine (Se-Met) and selenocystine (Se-Cys2), activating selenoenzymes and eliminating the excessive ROS (Figure 3). Recently, in our other Se-related work, we paid attention to the chiral nanomaterials and fabricated a chiral glutathione (GSH) SeNPs (G@SeNPs), coated with GSH on the surface of SeNPs, capable of preventing oxidation damage caused by palmitic acid (Huang et al., 2020). G@SeNPs showed ROS-responsive and clearance activities in INS-1 cells. Positron emission tomography imaging of chiral G@SeNPs *in vivo* illustrated that the ^64^Cu-labeled l-G@SeNPs were cleared slower in organs than d-G@SeNPs because of the homologous adhesion between l-GSH and the l-phospholipid membrane. This remaining higher concentration of l-G@SeNPs contributed to the stronger antioxidant activities.

Zhang et al. (2015) developed a biocompatible ROS-responsive β-cyclodextrin (β-CD) NPs through the conjugation of 4-phenylboronic acid pinacol ester (PBAP) onto the hydroxyl groups of β-CD for upgraded drug delivery applications. Because of the sensitivity of boronic ester units to the oxidation-responsive microenvironment, the newly obtained (Ox-bCD) material is hydrolyzed into parent β-CD molecules when exposed to ROS, displaying superior biocompatibility both *in vitro* and *in vivo*. Additionally, other therapeutics, such as imaging agents and biomacromolecules, can also be transported using this ROS-triggered nanocarrier for different applications.

Proteins have also been used as carriers for ROS-responsive cancer therapy. For instance, a protein-based delivery system named RNase A-NBC is responsive to ROS designed through a convenient chemical conjugation of 4-nitrophenyl 4-(4,4,5,5-tetramethyl-1,3,2-dioxaborolan-2-yl) benzyl carbonate (NBC) with the lysine residues of RNase A. These RNase A-NBC NPs present with minor cytotoxicity against normal cells but selective inhibition cytotoxicity against tumor cells because of the high concentration of ROS in malignant cells compared with the healthy cells. The high levels of H_2_O_2_ react with the amide bond and induce the lysine deprotection in cancer cells, reestablishing the cytotoxicity effect of RNase A in tumor cells. These protein-based pharmaceutical products are used as a tool targeting ROS-responsive cancer therapy (Wang et al., 2014).

**Figure 4:**
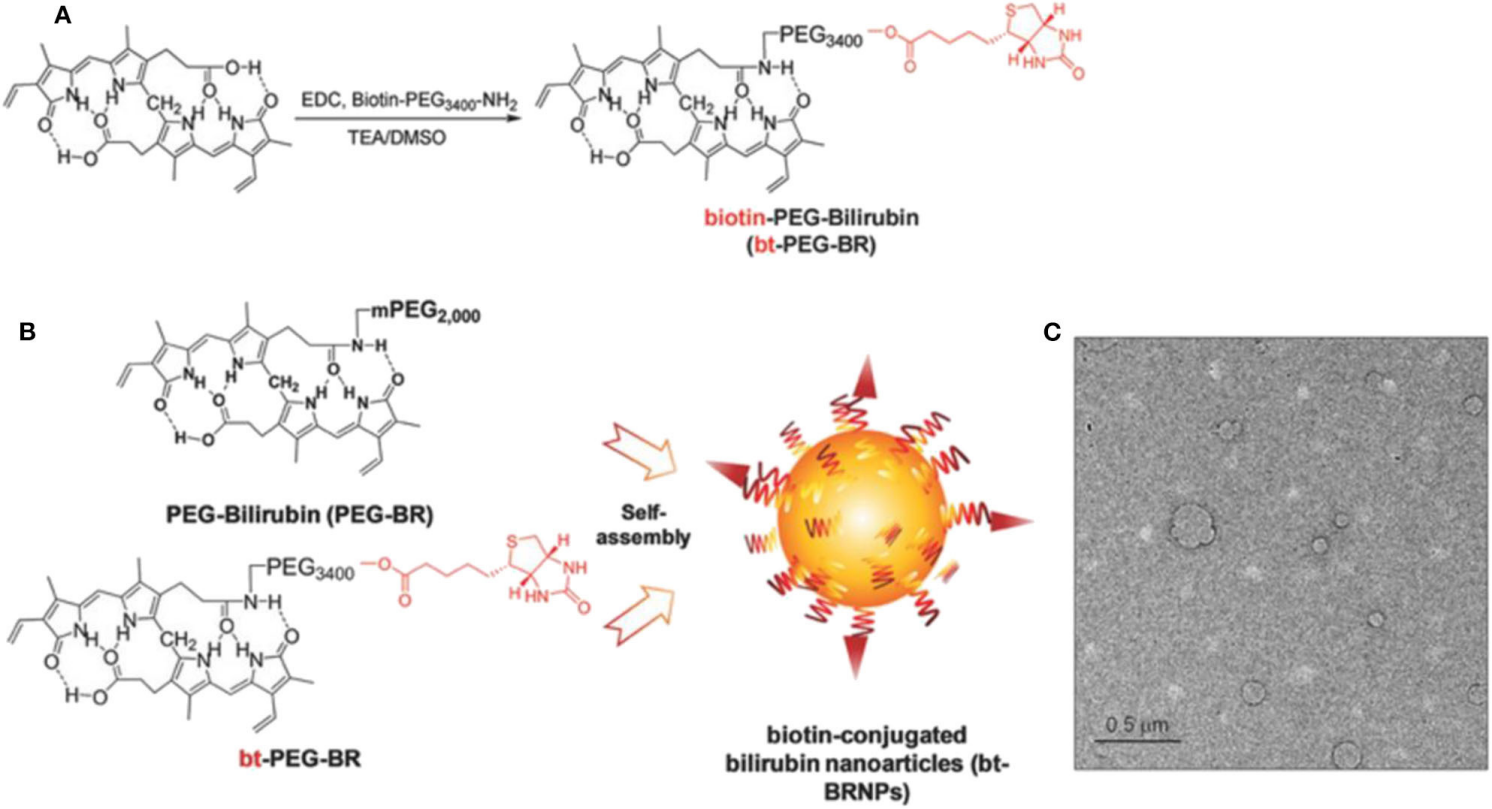
Biotin-conjugated bilirubin nanoparticles (bt-BRNPs) are formed from biotin-PEG-bilirubin (bt-PEG-BR) and PEG-bilirubin (PEG-BR) (Lee et al., 2018) (Copyright 2018, reproduced with permission from Elsevier). **(A,B)** Scheme for the synthesis of bt-PEG-BR starting from free bilirubin and biotin-PEG **(A)** and the formation of bt-BRNPs by self-assembly from PEG-BRs and bt-PEG-BR in PBS **(B)**. **(C)** Transmission electron microscopy (TEM) images of bt-BRNPs. Scale bar: 500 nm.

ROS is elevated in the tumor microenvironment and the biotin transporter; avidin is overproduced in many tumors. The cancer-targeting ROS-responsive nanocarriers release the drugs into the tumor microenvironment, resulting in higher antitumor efficacy. Based on the cancer-targeting and ROS-responsive dual concepts, Lee et al. (2018) proposed new bilirubin-based NPs (BRNPs) using biotin as cancer targeting ligand and bilirubin as the ROS-responsive carrier. Additionally, doxorubicin (Dox) is loaded as a therapeutic drug. In the synthesis of this drug, bt-PEG-BR is first obtained by reacting bilirubin and biotin-PEG, and then the Dox@bt-BRNPs are prepared in a single-step self-assembly procedure, with its size ~100 nm (Figure 4). Dox is released from the BRNPs after incubation with a peroxy radical precursor, 2,2′-azobis (2-amidinopropane) dihydrochloride, exhibiting the ROS-responsive releasing activities. Dox@bt-BRNPs has superior anticancer efficacy in biotin transporter–overexpressing HeLa cells than the free Dox. Similar results have been reported in xenograft mice. More BRNPs are preferentially accumulated and distributed in tumor areas than in other organs, as reported via *in vivo* fluorescence imaging assays (Lee et al., 2018). The biodegradability and biocompatibility of the bt-BRNPs made BRNPs as novel ROS-responsive nanocarriers for treating various tumors effectively.

### ROS/GSH-Responsive Nanocarriers for Cancer Therapy

Compared with healthy cells, malignant cells have a strong reduction environment because of the excessive production of intracellular GSH. However, some cancer cells produce excessive ROS simultaneously, resulting in increased oxidative stress (Fang et al., 2009). Besides, regarding the redox potential difference, cancer cells are characteristically heterogeneous. The levels of GSH/ROS vary across different stages of tumor growth and reproduction, and excessive production of ROS and GSH is present in various cancers or different areas of the same malignancy at the same time (Marusyk and Polyak, 2010). Nanocarriers capable of a dual response to ROS/GSH have attracted broad interests because of their application prospects in controllable packaging and drug delivery in physiological environments.

**Figure 5:**
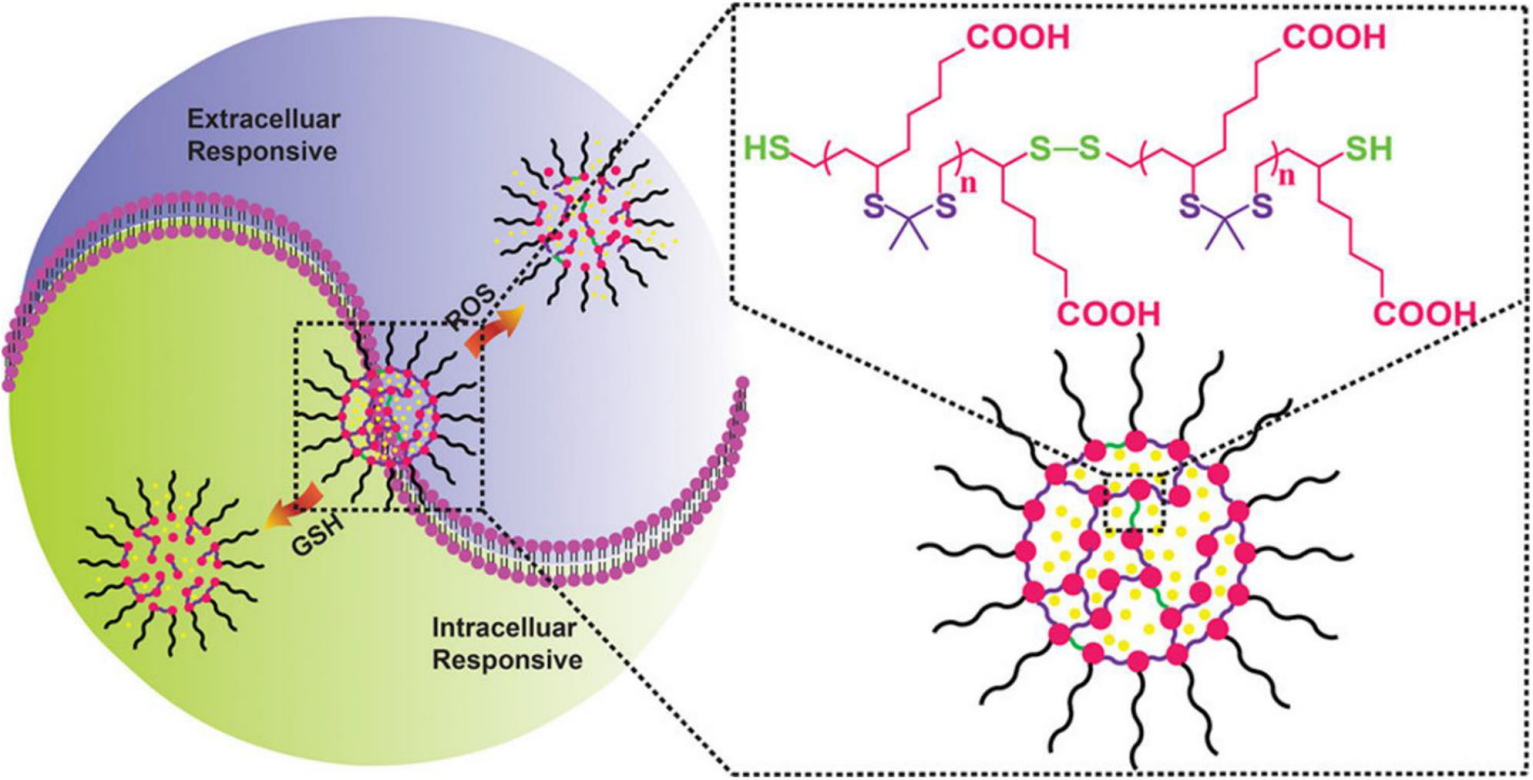
Schematic illustration of the ROS and GSH dual-responsive nano-DDS, the structure of nano-DDSs, containing both ROS-responsive (purple) and GSH-responsive (green) motifs (Chen et al., 2018) (Copyright 2018, reproduced with permission from American Chemical Society).

Luo et al. (2016) reported a new redox dual-reaction prodrug nanosystem self-assembled from paclitaxel (PTX), oleic acid (OA), and thioether bonds. This novel prodrug nanosystem provides a solution to issues associated with the low drug loading and low-efficiency drug release of PTX hydrophobic drugs and has been used for additional drug development. PTX is released via thiolysis by GSH or oxidation by ROS and exhibits potent *in vivo* antitumor efficacy in KB-3-1 tumor mice, without non-specific toxicity to major organs and tissues (Luo et al., 2016). This redox dual-sensitive polymers or complexes offer effective anticancer drug delivery possibilities. In the work of Chen et al. (2018), a type of thioketal NPs (TKNs) with double reactivity to H_2_O_2_ and GSH was designed for PTX delivery. This dual-responsive nanocarrier is sensitive to biologically relevant levels of GSH, and H_2_O_2_, releases drugs on demand and is biodegraded into biocompatible by-products after completing drug delivery tasks, compared with other stimuli-responsive nanocarriers (Figure 5). Given the variability in redox potential gradients across different microenvironments *in vivo*, the TKNs loaded with PTX (PTX-TKN) respond first to extracellular ROS, followed by the intracellular GSH, to achieve the controlled release of PTX into tumor sites. Both *in vitro* and *in vivo* findings showed that PTX-TKN is selective for cancer cells with high ROS and GSH levels.

### Applications of ROS-Responsive Nanocarriers in Combination Other Therapy Strategies

Chemotherapy, combined with hyperthermia, has attracted considerable research attention in disease treatment, such as cancer. Photothermal therapy (PTT) has represented an extraordinary non-invasive approach for cancer treatment, and photothermal agents are able to covert near-infrared (NIR) light into thermal energy under light irradiation. The NIR lasers most commonly used in PTT are 808 and 980 nm, and the safe power density limits are ~0.33 and ~0.726 W cm^−2^, respectively. Precise delivery of drugs to complicated and specific pathological sites while controlling the quantitative release of drugs remains challenging. Therefore, to ensure the simultaneous delivery of chemotherapeutic drugs and photothermal agents to the tumor area and achieve their synergistic effect, Xiao et al. (2015) developed a thermal and ROS dual-responsive polymer with an alternating structure of hydrophilic and hydrophobic links in its backbone. The triblock copolymer is easily synthesized via thiophene polymerization of the poly(ethylene glycol) (PEG) diacrylate and 1,2-ethanedithiol (EDT) monomer. Nile red is effectively encapsulated into the core of the nanocarriers at physiological temperatures and is released upon the destruction of the NP triggered by oxidation. This adjustable thermal response behavior combined with oxidizable thioether groups renders these PEG-EDT copolymers as promising ROS-reactive drug delivery system. Moreover, Wang and coworkers fabricated a NIR light and ROS dual-responsive Se-inserted copolymer (I/D-Se-NPs) for synergistic thermo-chemotherapy (Wang et al., 2017). The photothermal agent (ICG) and the chemotherapeutic drug (Dox) are loaded. A 785-nm irradiation at 1.0 W cm^−2^ is used to evaluate the photothermal conversion of I/D-Se-NPs. The ΔTm value of I/D-Se-NPs was 7.8°C when the ICG was used at a dose of 2.0 μg mL^−1^, exhibiting promising photothermal conversion efficiency.

**Figure 6:**
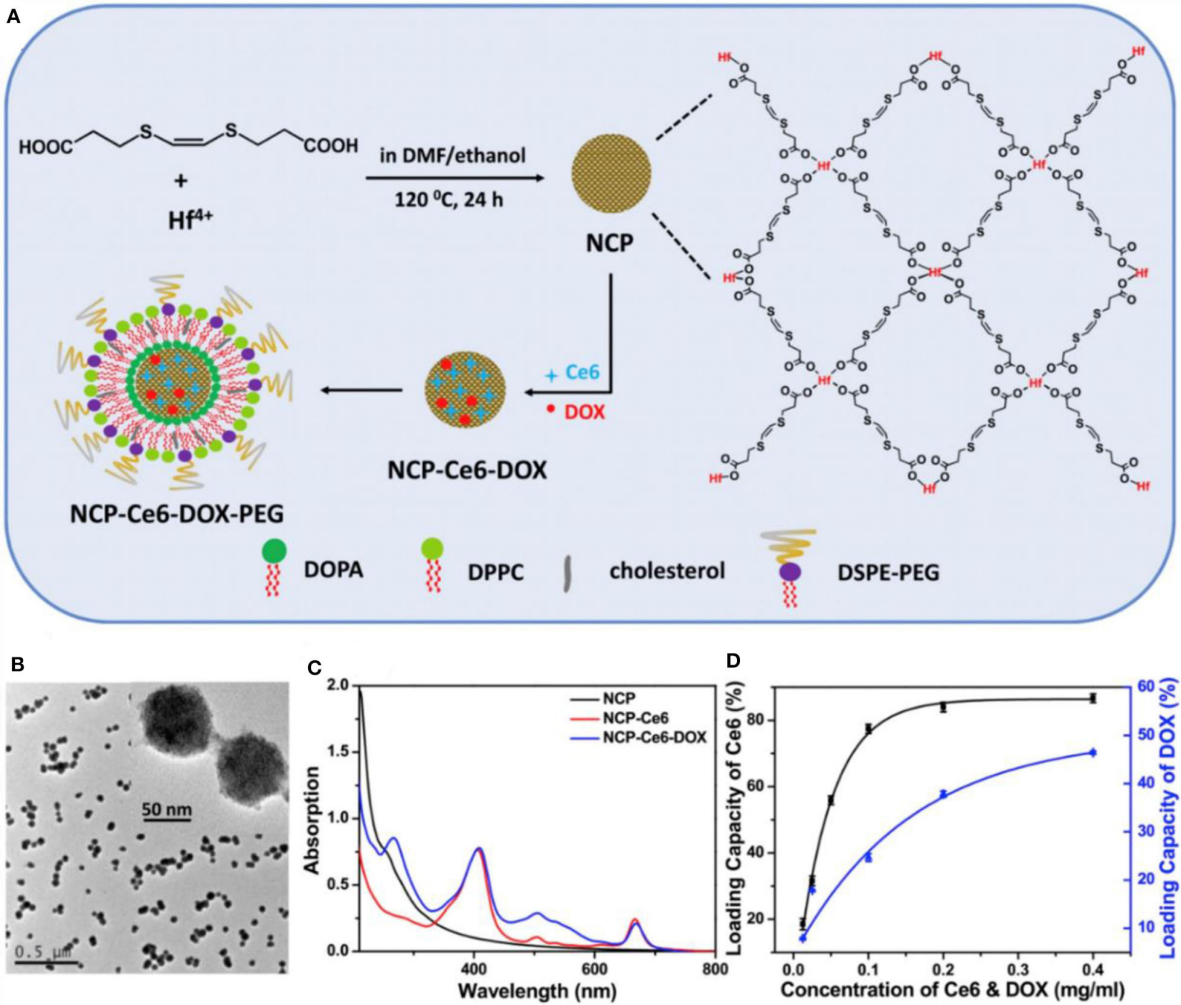
The synthesis and characterization of NCP-Ce6-DOX-PEG nanoparticles (Liu et al., 2017a) (Copyright 2017a, reproduced with permission from Elsevier). **(A)** The schematic illustration for the synthesis of NCP-Ce6-DOX-PEG nanoparticles. **(B)** A TEM image of NCP nanoparticles. The insert is an image with higher resolution. **(C)** UV-vis-NIR spectra of NCP, NCP-Ce6, and NCP-Ce6-DOX. **(D)** Quantification of Ce6 and DOX loadings at different feeding concentrations of Ce6 and DOX in ethanol. NCP solutions with the same concentration (0.05 mg/mL) were used in this experiment.

The combination of ^1^O_2_-responsive nanocarriers with other treatment, such as photodynamic therapy (PDT), has remarkable synergistic therapeutic effects (Wang et al., 2016; Yang et al., 2016; Yu et al., 2016; Li et al., 2017b; Liu et al., 2017a). In PDT, non-toxic photosensitizers are activated by exogenous light of a specific wavelength to transfer their excited energy to the surrounding oxygen to produce ROS. Ce6 is extensively used in photodynamic treatment of cancer as a photosensitizer and effectively generates ^1^O_2_ under light irradiation. Yang and coworkers developed a smart mesoporous silica nanorod drug delivery system with a photosensitizer chlorin e6 (Ce6) doped in a *bis*-(alkylthio) alkene (BATA) as the nanocarrier, named CMSNRs. The BATA linker is cleaved by ^1^O_2_ produced by Ce6 after illumination with 660 nm light irradiation, Dox is released from this ^1^O_2_-responsive CMSNRs both *in vitro* and *in vivo* (Yang et al., 2016). Furthermore, Liu et al. (2017a) developed light-controlled, ^1^O_2_-responsive polymers, NCP-Ce6-DOX-PEG, through a solvothermal method with a size of about 70 nm (Liu et al., 2017a). UV-vis-NIR absorption spectrum showed that the Ce6 and Dox were loaded in this nanocarrier with the loading ratio of 80% of Ce6 (Figure 6). As low as 5 mW cm^−2^ light irradiates the release of ^1^O_2_, breaking the BATA link. This accelerated ^1^O_2_-responsive nanoscale coordination polymers in tumors are observed through computed tomography imaging, and this combination of chemophotodynamic therapy has excellent antitumor efficacy *in vitro* and *in vivo* (Liu et al., 2017a).

**Figure 7:**
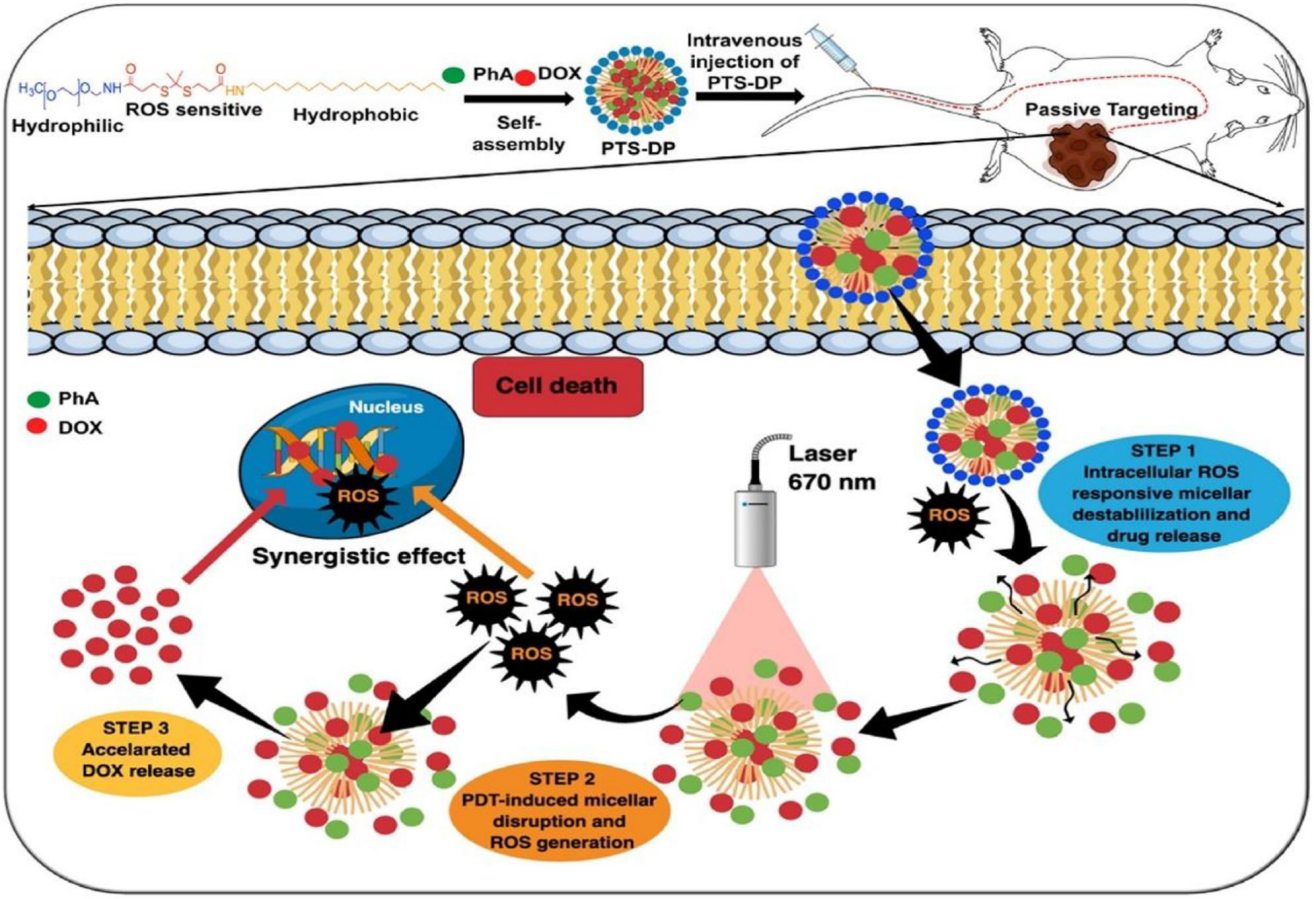
Schematic illustration of ROS cascade–responsive drug release of PTS-DP for enhanced locoregional chemophotodynamic therapy (Uthaman et al., 2020) (Copyright 2020, reproduced with permission from Elsevier).

Sun et al. (2019) designed photoactivatable photodynamic PEG-coated drug nanoplatforms for core-shell cooperative chemotherapy and PDT. A new type of photodynamic polymer was rationally developed and synthesized through conjugation of pyropheophorbide-a (PPa) with PEG 2000 (PEG2k). In this system, PTX is encapsulated as the therapeutic drug, and PPa is utilized as the hydrophobic and photodynamic part of the amphiphilic PPa-PEG2k polymer. PPa-PEG2k is used in PDT treatment; under laser irradiation, PPa-PEG2k produces ROS and synergistically promotes endogenous ROS generation in cancer cells to promote PTX release. Nanomicelles have also been employed in the construction of a photoactivatable system. A recent study on new nanomicelles constructed long-circulating photoactivated nanocarriers via self-assembly of thioketal and a PEG-stearyl amine conjugate (PTS) (Uthaman et al., 2020). Dox and photosensitive pheophorbide A (PhA) are coloaded into the formed nanocarriers to enhance local chemical and PDT (Figure 7). The resulting Dox- and PhA-loaded nanocarriers exhibit ROS stimulus responsiveness after accumulating in the tumor area to release the internally loaded DOX and PhA. Moreover, after laser irradiation of the tumor area, PhA initially released into the tumor produces enhanced ^1^O_2_, thereby promoting the rapid dissociation of nanocarriers and accelerating the release of DOX. ROS triggers the photoactivated PhA to release Dox, which increases local ROS levels gradually to inhibit cancer cell growth and enhance antitumor immunity synergistically. Therefore, the combination of ROS-sensitive PTS nanocarriers with local chemical PDT is a promising method for treating tumors.

### Application of ROS-Responsive NPs for Inflammation Treatment

**Figure 8:**
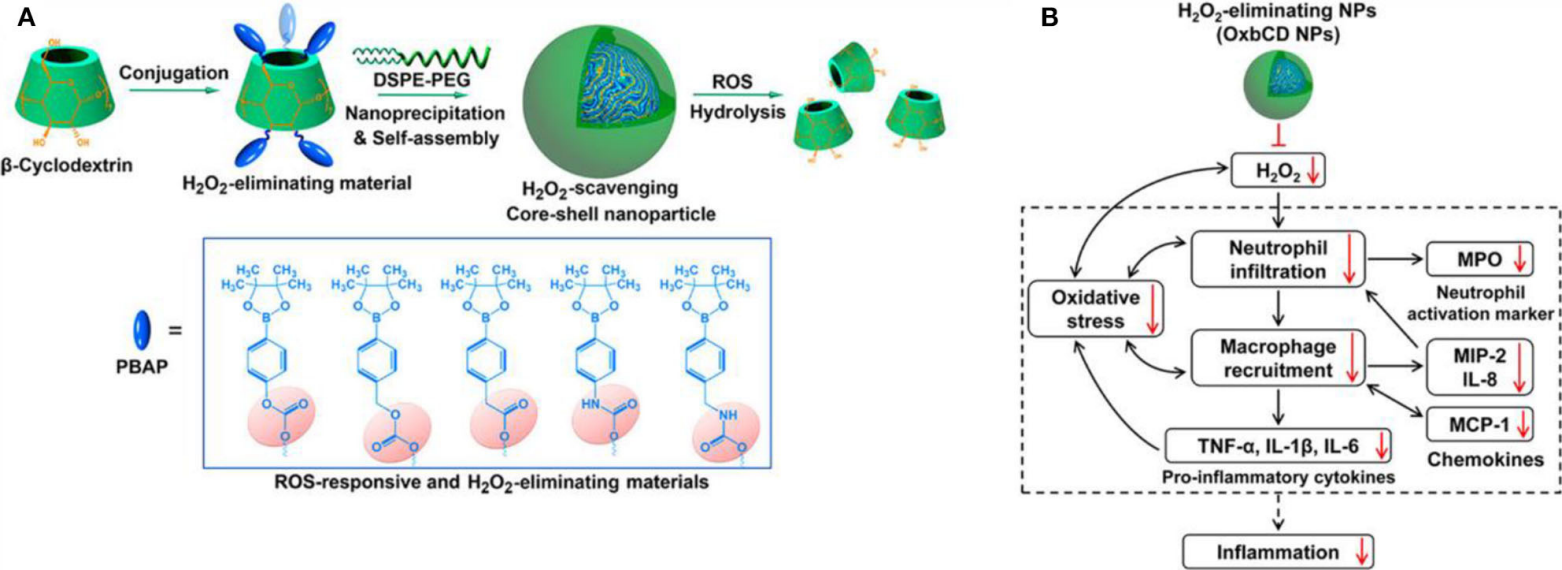
**(A)** Design and synthetic routes of different H_2_O_2_-scavenging materials derived from β-CD, as well as engineering of anti-inflammatory nanoparticles. **(B)** Schematic illustration of anti-inflammatory mechanisms of H_2_O_2_-eliminating OxbCD NPs (Zhang et al., 2017) (Copyright 2017, reproduced with permission from American Chemical Society).

**Figure 9:**
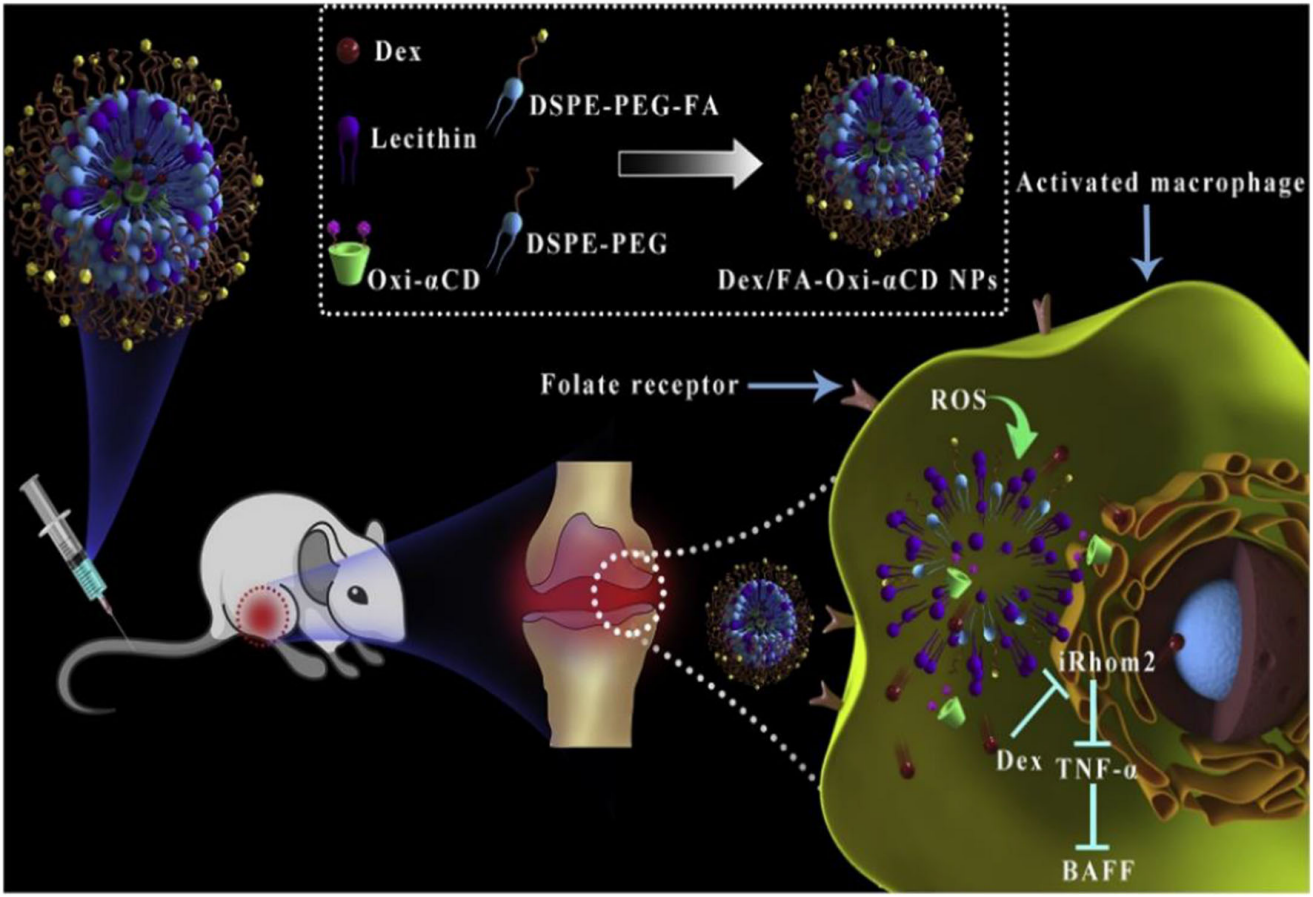
Schematic illustration of Dex-loaded ROS-responsive NPs for targeted RA therapy. The proposed mechanisms showing a cascade of events in macrophages via the iRhom2–TNF-α-BAFF signaling pathway (Ni et al., 2020) (Copyright 2020, reproduced with permission from Elsevier).

In recent years, preclinical and clinical research has demonstrated that excessive ROS at the inflammatory site accelerates disease progress. Numerous studies utilize ROS as triggers in developing ROS-responsive NPs carrying anti-inflammatory drugs (Pu et al., 2014; Feng et al., 2016; Zhang et al., 2017, 2020; Chen et al., 2019; Li et al., 2019, 2020; Ni et al., 2020). The release of loaded drugs in the inflammatory joints improves patient symptoms. Boronic esters are excellent and selective H_2_O_2_-responsive units and are degraded under physiologically relevant H_2_O_2_ levels. Furthermore, these types of boronic ester–functionalized nanomaterials possess good safety profiles. They are a promising approach for the development of ROS-responsive nanocarriers with significant potential for clinical translation. Zhang et al. (2017) designed and synthesized a series of ROS-responsive core-shell OxbCD NPs via conjugation of PBAP groups onto a β-CD with H_2_O_2_-eliminating profiles (Figure 8A). These OxbCD NPs have excellent antioxidant and anti-inflammatory activities. The anti-inflammatory mechanisms of OxbCD NPs are shown in Figure 8B. OxbCD NPs reverse the oxidative stress and repress cell death triggered by H_2_O_2_ in RAW264.7 cells. OxbCD NPs efficaciously decrease the secretion of the classic inflammatory chemokines, such as MCP-1, MIP-2, and IL-8, as well as the proinflammatory cytokines consisting of tumor necrosis factor α (TNF-α), IL-1β, and IL-6, the expression levels of which are high in H_2_O_2_ treatment macrophages. Additionally, neutrophil infiltration and macrophage recruitment are inhibited with the treatment of OxbCD NPs. Moreover, OxbCD NPs suppress the expression of the activation marker, MPO, and other oxidative mediators. Finally, OxbCD NPs loaded with anti-inflammatory drugs have superior efficacy as seen in an acute inflammation model of peritonitis in mice. RA is an immune-mediated inflammatory disease with higher levels of ROS. ROS in arthritis tissues leads to the overproduction of the cytokines consisting of TNF-α, IL-1β, and IL-6. The interactions between these inflammatory factors and ROS mainly contribute to the acceleration of RA progression. Ni et al. (2020) developed ROS-responsive dexamethasone (Dex)-loaded NPs named Dex/folic acid (FA)-Oxi-αCD using α-cyclodextrin (α-CD) as nanocarriers and FA as targeting group for the treatment of RA (Figure 9). Dex/FA-Oxi-αCD is sensitive to H_2_O_2_, and elevated levels of H_2_O_2_ promote the degradation of Oxi-αCD, releasing Dex. An anti-inflammatory mechanism study revealed that Dex/FA-Oxi-αCD inhibits the expression of iRhom2, TNF-α, and BAFF *in vitro* and *in vivo*. FA modification accumulates the biodistribution of Dex/FA-Oxi-αCD in the inflamed joints of RA, and the therapeutic efficacy is significantly improved compared to free Dex (Ni et al., 2020).

**Figure 10:**
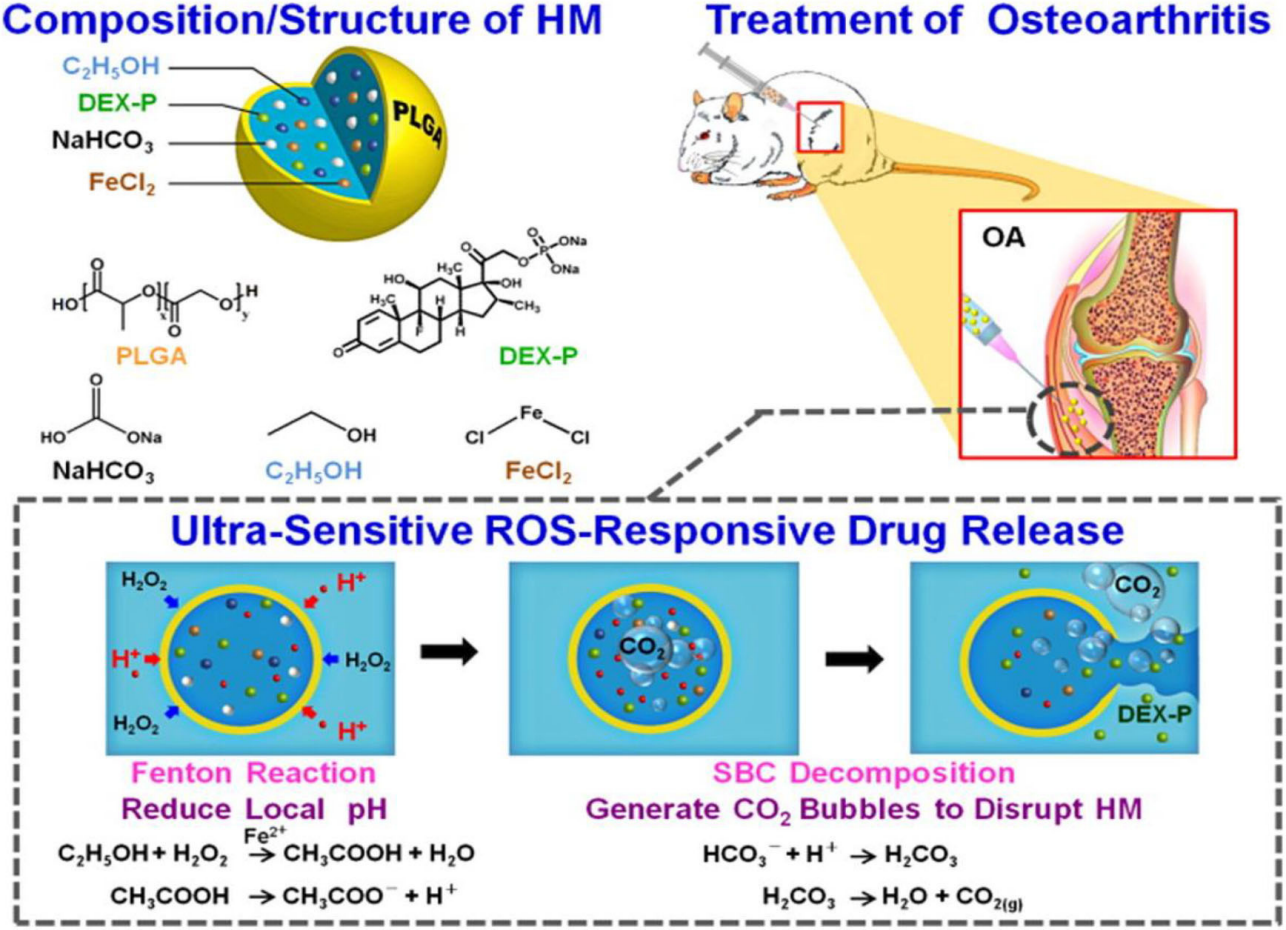
Composition/structure of the ultrasensitive ROS-responsive gas-generating HM developed herein and its mechanism in the treatment of OA (Chung et al., 2015) (Copyright 2015, reproduced with permission from American Chemical Society).

Chung et al. (2015) fabricated an inflammatory microenvironment ultrasensitive ROS-responsive gas-generating carrier for the treatment of osteoarthritis. In this work, the PLGA hollow microsphere (HM) carrier is functionalized with an anti-inflammatory drug, dexamethasone sodium phosphate (DEX-P); an acid precursor (composed of ethanol and FeCl_2_); and a bubble-generating agent, sodium bicarbonate (SBC). As shown in Figure 10, in the inflammatory environment, the encapsulated ethanol is oxidized by H_2_O_2_ in the presence of Fe^2+^ by the Fenton reaction, producing an acidic milieu. The decomposition of SBC in acidic conditions generates CO_2_ bubbles to disrupt the shell wall of HM, and the payload anti-inflammatory drug DEX-P is released in high dosage with potential efficacy against joint destruction (Chung et al., 2015).

### ROS/pH Response Nanocarrier for Inflammation Treatment

Where ROS is excessively produced, the pH changes. For example, compared to normal tissue/blood flow (pH ~7.4), the tumor/inflammation area is slightly acidic (pH 5.4–7.1). Much drug development work has aimed at designing nanoplatforms that respond to multiple stimuli to enhance the performance of nanoagents through improved delivery of drugs to the target site. Physiological pH gradients and ROS levels have been extensively utilized in the design of stimuli-responsive nanosystems to deliver drugs to target sites. These delivery systems are usually based on an NP that undergoes swelling, charge conversion, membrane fusion, or bond-breaking after receiving pH and ROS signals. Actively targeted NPs that can simultaneously respond to low pH and high concentrations of ROS are potential nanocarriers for the precise delivery of therapeutic drugs to the target site. pH/ROS dual-responsive nanocarriers are constructed by combining pH-sensitive materials and oxidation-responsive materials. Through adjusting the weight ratio of the pH-sensitive materials and oxidation-responsive materials, it is possible to adjust the pH/ROS response capability, thereby providing nanocarriers with different hydrolysis characteristics in an inflammatory microenvironment. Studies have shown that pH/ROS double-reactive NPs can be used as an effective and safe nanocarrier for the precise treatment of vascular inflammatory diseases.

**Figure 11:**
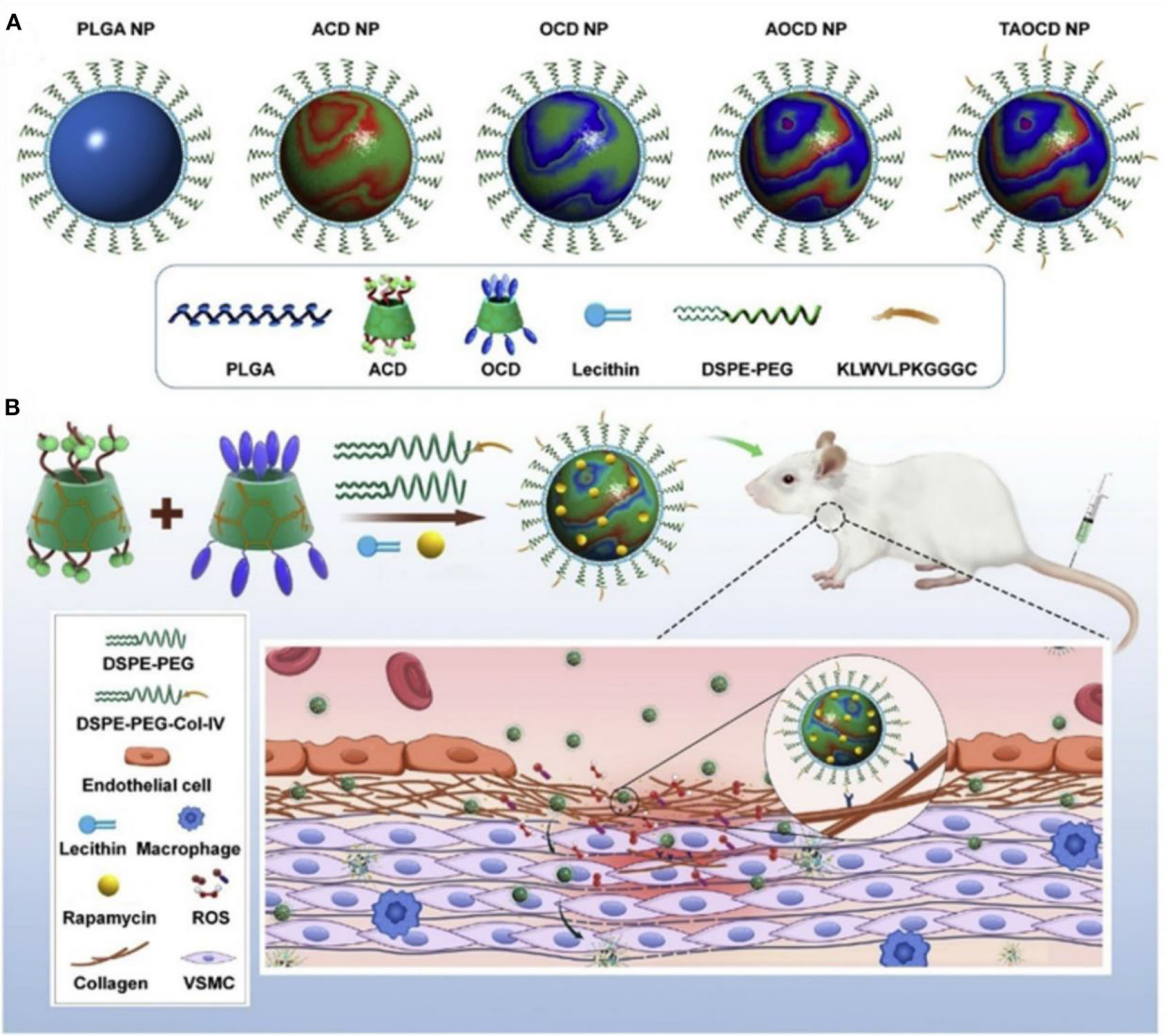
Design and engineering of pH/ROS dual-responsive nanotherapies for targeted treatment of restenosis (Zhang et al., 2020) (Copyright 2020, reproduced with permission from Elsevier). **(A)** Schematic illustration of different NPs examined in this study. **(B)** Engineering of a dual-responsive, targeting rapamycin nanotherapy based on a pH-sensitive β-CD material (ACD) and a ROS-responsive β-CD material (OCD), as well as targeted treatment of a vascular inflammatory disease of restenosis.

The inflammatory bowel disease (IBD) is characterized by high levels of ROS in the diseased sites, and oxidative stress is involved in and contributes to the pathogenesis and progression of IBD (Tian et al., 2017). Bertoni et al. (2018) synthesized phenylboronic ester–modified dextran (OxiDEX) NPs loaded with rifaximin (RIF) for targeted therapy of IBD, with a sequential responsive behavior to both pH and ROS. The permeability of OxiDEX NPs is remarkably lower compared with the traditional enteric formulation in an *in vivo* intestinal membrane mimicking the C2bbe1/HT29-MTX cell monolayer model. High amount of the drug is transported to the diseased sites, and the therapeutics efficacy is significantly improved, reducing unspecific absorption and systemic side effects. Lin et al. (2020) developed a polyadenylic acid micelle (PD-MC) based on the ROS and pH dual-sensitive block polymer PEG-P (PBEM-co-DPA). The micelles have excellent potential for improving the biocompatibility of resveratrol glycosides and effective targeted drug delivery into the liver fibrosis microenvironment. *In vitro* and *in vivo* studies show that PD-MCs inhibit inflammation and oxidative stress and reduce apoptosis of liver cells Notably, the empty micelle promotes liver ROS depletion at the pathological site; hence, it has an anti-inflammatory effect. Therefore, PD-MCs have great potential for clinical use in anti–liver fibrosis drug treatment methods. More recently, Zhang et al. (2020) designed a pH-sensitive β-CD material (ACD) and a ROS-responsive β-CD material (OCD) NPs with loaded rapamycin (RAP) for targeted treatment of vascular inflammatory diseases. These NPs were constructed by the combination of a pH-sensitive unit (ACD) and an oxidation-responsive unit (OCD) facilely synthesized via acetylation of β-CD (Figure 11). The loaded RAP molecule is released from the RAP/AOCD NP in high levels of H_2_O_2_ or low pH inflammatory microenvironment. IV collagen (Col-IV) is highly expressed in the inflammation sites, by a surface decoration of AOCD NP with a Col-IV–targeting peptide (KLWVLPKGGGC); the resulting peptide-modified targeted RAP/AOCD NP efficiently accumulates in the rat vascular smooth muscle cells (VSMCs) *in vitro*, as well as in the balloon-injured arteries of rats *in vivo*, and inhibits the migration and proliferation of VSMCs and the formation of neointimal. This shows potential antirestenosis effects (Zhang et al., 2020). Finally, this constructed cascade pH/ROS dual-responsive drug targeted delivery system (AOCD NP and RAP/AOCD) is safe *in vitro* and *in vivo* in long-term treatment experiments. AOCD NP is a potential novel tool for delivering drugs to the inflammatory diseased sites utilizing the ROS microenvironment.

### Application of ROS-Responsive NPs for Neurodegenerative Diseases

Most neurodegenerative diseases, including AD, PD, and ischemic stroke, are characterized by increased inflammation and ROS with cognitive decline and memory loss. Elevated ROS triggers inflammation, promoting the deterioration of diseases. Numerous studies have developed ROS-responsive drug delivery systems for treating neurodegenerative diseases by reducing the elevated levels of ROS (Li et al., 2018; Lv et al., 2018; Ballance et al., 2019; Jiang et al., 2019).

**Figure 12:**
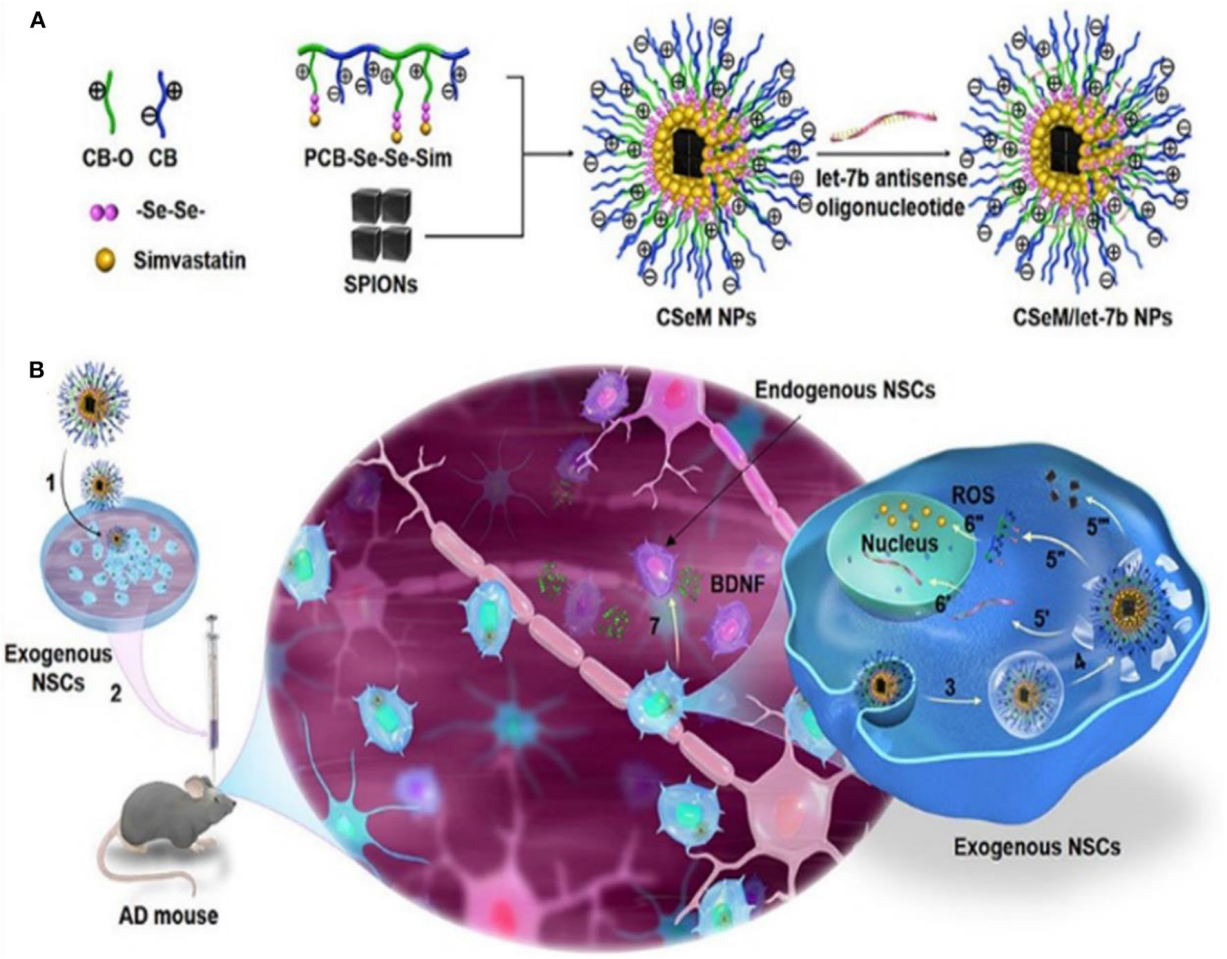
Illustration of strategy for construction and functionalization of the traceable CSeM/let-7b NPs (Li et al., 2018) (Copyright 2018, reproduced with permission from American Chemical Society). **(A)** Preparation of the traceable CSeM/let-7b NPs. Polyprodrug amphiphiles were self-assembled to load SPIONs and absorb let-7b antisense oligonucleotide. **(B)** Schematic illustration of the mechanism of the traceable CSeM/let-7b NPs controlling exogenous NSCs as BDNF source for AD therapy.

AD is among the most common neurodegenerative disorders in which high levels of ROS cause oxidative stress seen in patients with AD. ROS is an excellent therapeutic target in AD as demonstrated by experimental and clinical research findings (Behl et al., 1994; Butterfield and Lauderback, 2002; Geng et al., 2012; Li et al., 2013; Hu et al., 2015). ROS-responsive antioxidant nanotherapies with ROS-eliminating abilities have shown good clinical outcome in AD patients. Li et al. (2018) developed a self-assembled ROS-responsive positively charged polyprodrug amphiphiles by connecting poly(carboxy betaine) and simvastatin using a ROS-responsive diselenide bond (Figure 12). Simvastatin improves functional recovery of the spinal cord injury as seen in a rat model. It achieves this effect by upregulating the expression of brain-derived neurotrophic factors (BDNFs) leading to enhanced spatial memory recovery (Han et al., 2011). BDNF is an important neurotrophic factor that modulates nerve cell migration and neurogenesis, as well as stabilizes the intercellular environment (Zuccato and Cattaneo, 2009; Jiang et al., 2010). A NSC differentiation–promoting negative drug molecule, a lethal-7b antisense oligonucleotide (let-7b) (Zhao et al., 2010), and hydrophobic superparamagnetic iron oxide nanocubes (SPIONs), which is used for tracking mesenchymal stem cells (Park et al., 2017), are encapsulated in this polyprodrug amphiphiles to synthesize PCB-Se–Se-Sim/SPIONs/let-7b antisense oligonucleotide NPs (CSeM/let-7b NPs). Neural stem cells treated with CSeM/let-7b NPs show a remarkable improvement in memory function as seen in 2xTg-AD mice. This NP enhances the secretion of BDNF, yielding remarkably therapeutic effects *in vivo* (Li et al., 2018). Besides, CSeM/let-7b NP helps to trace the transplantation site and the migration of exogenous NSCs because of its high *r*_2_ value of SPIONs in magnetic resonance imaging.

**Figure 13:**
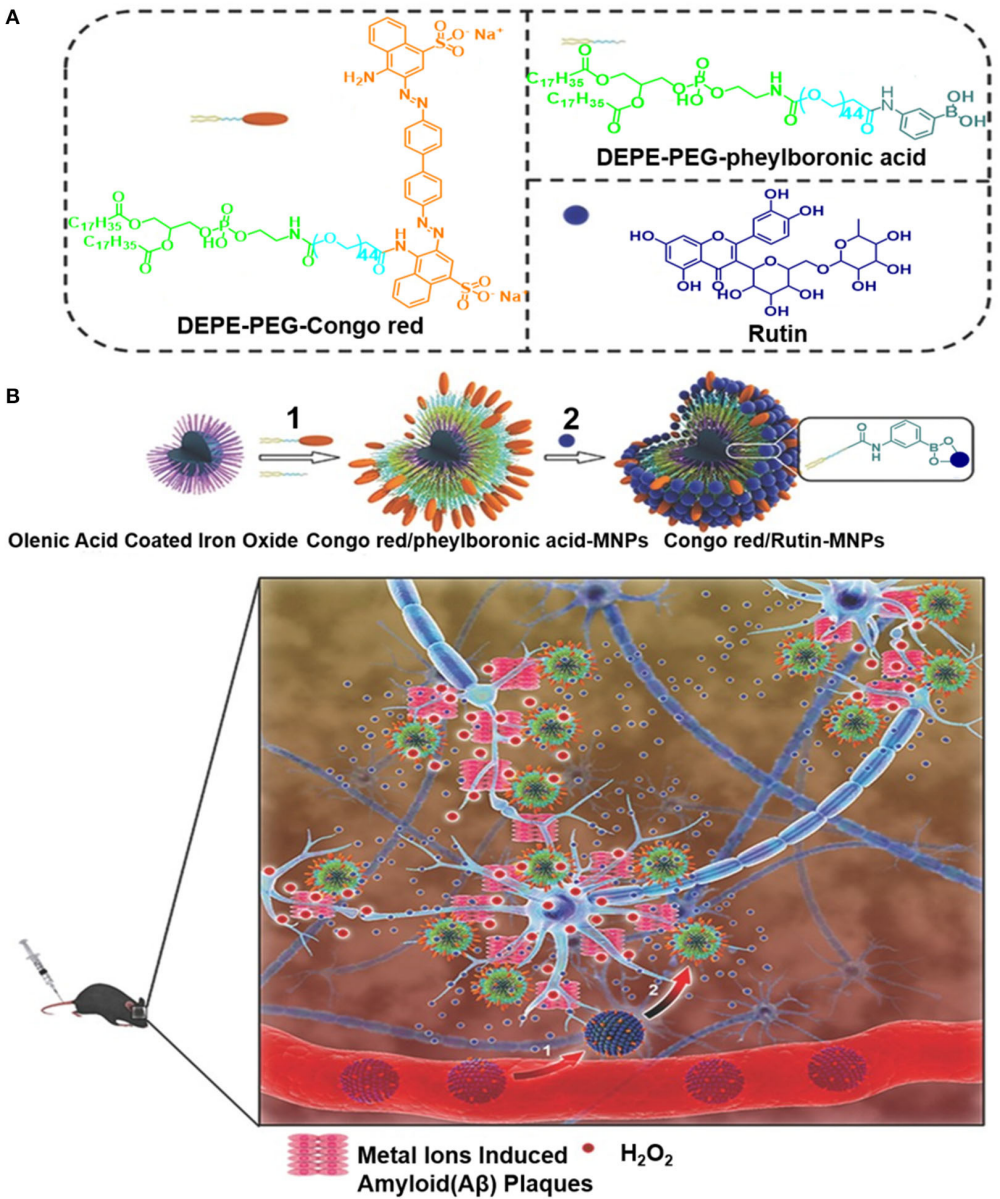
**(A)** The preparation of Congo red and Rutin–loaded magnetic nanoparticles (Congo red/Rutin-MNPs): (1) DSPE-PEG-Congo red and DSPE-PEG-phenylboronic acid were used to improve the biocompatibility of magnetic nanoparticles through a micelle formation procedure. (2) Rutin was grafted onto the surface of the nanoparticles through the formation of a boronate ester bond between vicinal diols and phenylboronic acid. **(B)** Schematic interpretation of Congo red/Rutin-MNPs *in vivo*: (1) Congo red/Rutin-MNPs coinjected with mannitol penetrated the BBB. (2) Congo red/Rutin-MNPs detected amyloid plaques specifically, realized targeted delivery, and controlled release of Rutin by H_2_O_2_ (Hu et al., 2015) (Copyright 2015, reproduced with permission from John Wiley and Sons).

Elsewhere, a spherical-like Congo red/rutin-MNPs nanotheranostic comprising a central Fe_3_O_4_ NP, the surface of which is coated with Congo red and rutin, was used to design a biocompatible H_2_O_2_-responsive magnetic nanocarrier for AD therapy (Hu et al., 2015). As illustrated in Figure 13, the biocompatibility of the Congo red/rutin-MNPs is improved by coating the DSPE-PEG-Congo red and DSPE-PEG-phenylboronic acid on the surface of Fe_3_O_4_ NPs. This carrier delivers high amount of the drug into the central nervous system using the PEGlyated modification, reducing its uptake by the reticuloendothelial system. The boronate ester bond between the vicinal diols and phenylboronic acid is cleaved using H_2_O_2_, and rutin is released from the Congo red/rutin-MNPs in an H_2_O_2_-responsive and concentration-dependent manner, thereby curtailing the effects of Aβ-induced cytotoxicity in SH-SY5Y cells and oxidative stress. Furthermore, the ultrasmall size of the Congo red/rutin-MNPs enables the detection of distribution of amyloid plaques and makes particles easier to cross the blood–brain barrier (BBB), rescuing memory deficits and neuronal loss in APPs we/PS1dE9 transgenic mice (Hu et al., 2015). The targeted delivery and controlled release properties of these Congo red/rutin-MNPs open up a new direction for application of theranostics in AD.

**Figure 14:**
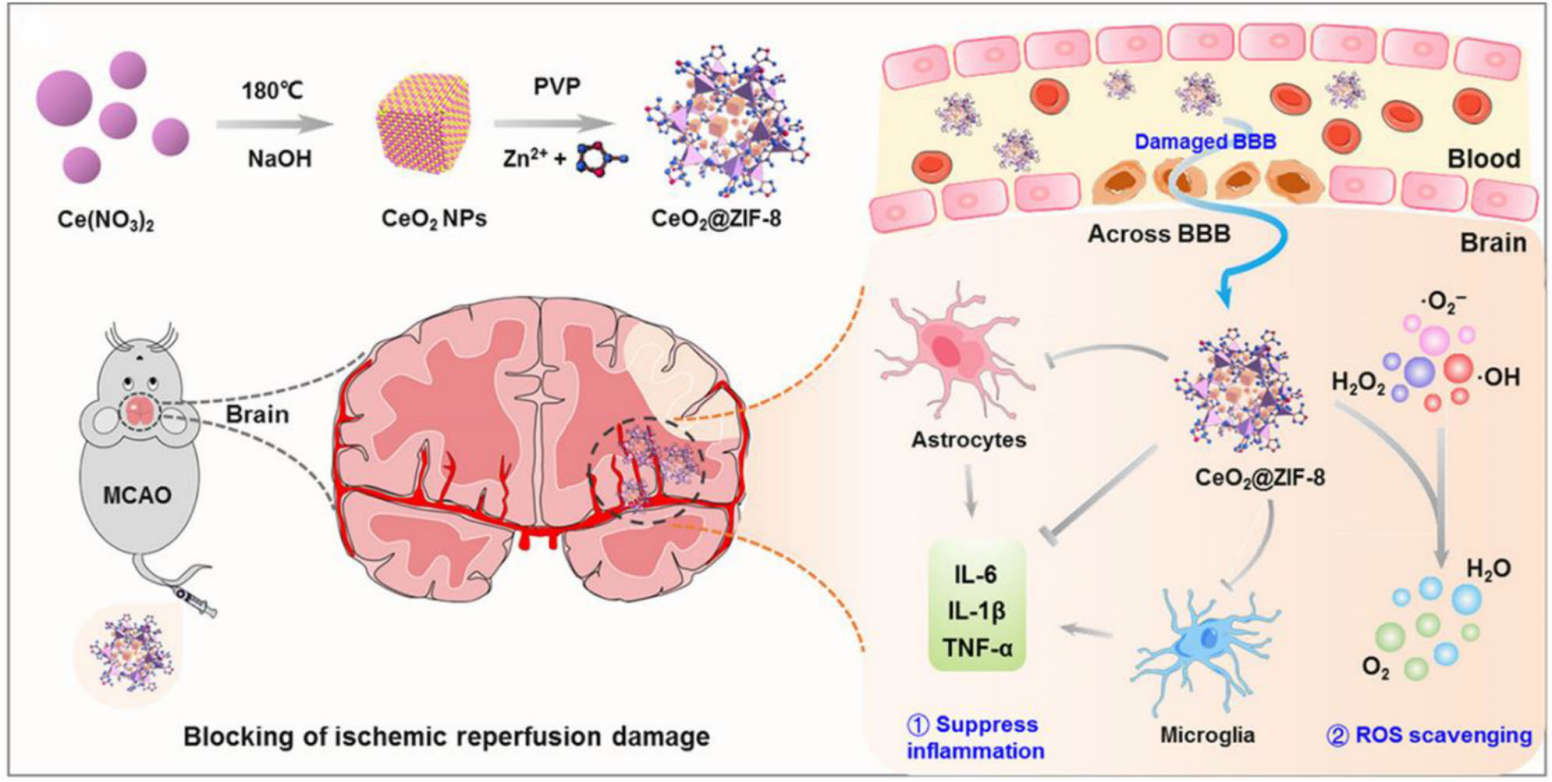
Schematic illustration for *in situ* synthetic approach of CeO_2_@ZIF-8 nanotherapeutics and its neuroprotective application mechanisms against reperfusion-induced injury in ischemic stroke (He et al., 2020) (Copyright 2020, reproduced with permission from American Association for the Advancement of Science).

Ischemic stroke is another neurodegenerative disease that causes long-term disability and death worldwide due to overproduction of ROS. High levels of ROS cause detrimental effects on neurons and tissue injury at ischemic sites (Benjamin et al., 2017). Therefore, reducing oxidative stress is a prospective therapeutic approach for ischemic stroke (Panagiotou and Saha, 2015; Amani et al., 2017; Liu et al., 2017b; Lv et al., 2018; He et al., 2020; Tapeinos et al., 2020). Indeed, many ROS-responsive nanocarriers have been developed for the treatment of ischemic stroke (Lu et al., 2016, 2019; Jiang et al., 2019). Recently, we developed bifunctional nanomaterials zeolitic imidazolate framework-8-capped ceria NPs (CeO_2_@ZIF-8 NPs) based on *in situ* synthesis strategy with ROS response and clearance capabilities (He et al., 2020). In this nanosystem, CeO_2_ NPs was first created as the core structure of the NPs through a facile hydrothermal method, which is coated with a ZIF-8 shell, regulating the size, shape, and surface charge of CeO_2_ inner core through the addition of polyvinylpyrrolidone. CeO_2_@ZIF-8 NPs is decomposed by H_2_O_2_, releasing CeO_2_ slowly, which exhibits effective ROS-scavenging activities *in vitro* and protects against tert-butyl hydroperoxide (t-BOOH)–induced PC-12 cytotoxicity (Figure 14). A pharmacokinetic study demonstrated that CeO_2_@ZIF-8 NPs were able to across BBB and have prolonged circular behavior in the blood, which enhances its accumulation in brain tissue with better therapeutic efficacy. Furthermore, CeO_2_@ZIF-8 NPs can effectively inhibit the activation of astrocytes and microglia and reduce the expression levels of inflammatory factors and lipid peroxidation in a middle cerebral artery occlusion (MCAO) injury ischemic rat model. Particularly, CeO_2_@ZIF-8 reduces brain damage in ischemic stroke rats with good *in vivo* biocompatibility and biosafety.

**Figure 15:**
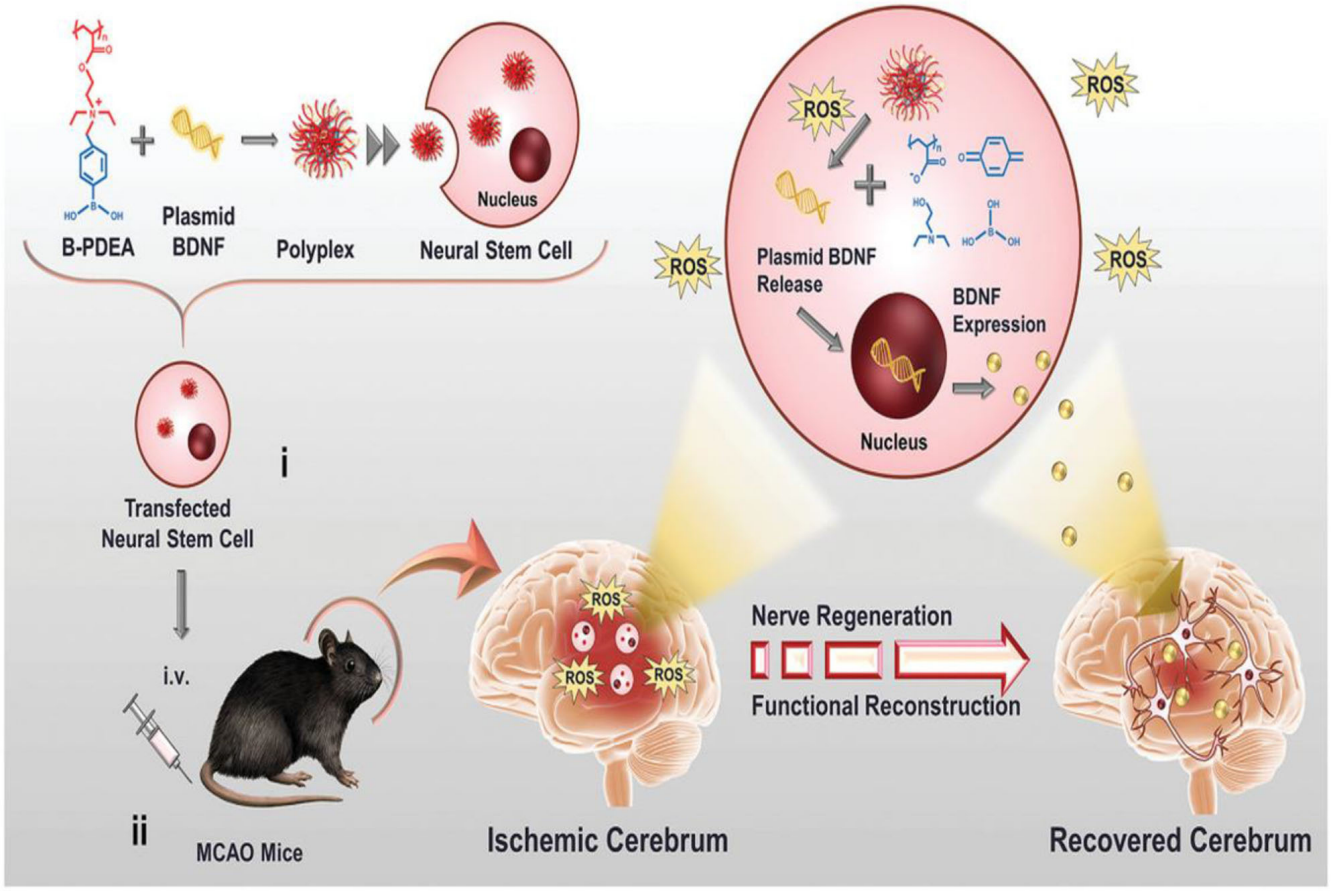
Schematic illustration of B-PDEA–mediated BDNF transfection of NSCs for ischemic stroke treatment (Jiang et al., 2019) (Copyright 2019, reproduced with permission from John Wiley and Sons). (i) B-PDEA complexes BDNF plasmids to form polyplexes used for transfection of NSCs. (ii) The transfected NSCs i.v. transplanted to MCAO mice migrate to the injured area in the brain, whose high level of ROS efficiently triggers B-PDEA's conversion to negatively charged polyacrylate and the release of BDNF plasmids for NSCs to express and excrete BDNF. The released BDNF stimulates nerve regeneration and functional reconstruction, giving rise to a significant therapeutic effect in ischemic stroke.

Endogenous NSCs are ischemia-homing elements that induce the production of extracellular matrix molecules, such as BDNFs to support neural cell growth (Aizman et al., 2009). Recently, Jiang et al. (2019) fabricated the first charge-reversal polymeric vector-transfected NSC with ROS responsiveness that homes the ischemia regions for synergistic ischemic stroke treatment (Figure 15). In this study, cationic poly [(2-acryloyl) ethyl (*p*-boronic acid benzyl) diethyl ammonium bromide] (B-PDEA) was first used to absorb plasmid DNA to form spherical polyplexes with excellent stability in a gel electrophoresis experiment. The newly constructed polyplexes effectively transfect NSCs via clathrin-mediated endocytosis with high gene transfection efficiency and less toxicity. After internalization into the cytosol, B-PDEA is first conversed to negatively charged polyacrylic acid by intracellular ROS. The released BDNF plasmids induce the NSCs to secret a high amount of BDNF into the injured ischemic cerebrum area in MCAO mice. Moreover, BDNF-NSCs eliminate the excessive ROS, resulting in the significant improvement of neurological and motor functions in MCAO mice (Jiang et al., 2019).

## Conclusions and Future Perspectives

Herein, we reviewed the role of ROS in various human diseases. Although ROS is important for normal functioning of the human body, excessive levels of ROS cause oxidative stress leading to the pathogenesis of diseases. The ROS-responsive nanocarriers used in scientific research and their biomedical applications in the treatment of diseases related to oxidative stress were discussed in this review. In the past decade, the rapid development in nanotechnology has expanded the types and preparation methods of ROS-responsive nanomaterials and nanocarriers, which have been applied in multiple biological systems. However, despite the considerable achievements made in the designing of ROS-responsive nanocarriers, the delivery efficiency of current drug carriers, controlled drug release profile, and *in vivo* therapeutic effects of these drug platforms remain unsatisfactory. Our current understanding of their therapeutic function and the underlying chemical/biological relationship remains preliminary, and our research on these nanocarriers is insufficient to guarantee commercialization. We have not seen the commercialized clinical application of ROS-responsive nanocarriers due to the complexity of majority of ROS-responsive nanocarriers; the manufacturing process, reproducibility, and quality are difficult to control. Several limitations hinder further clinical translation of these nanocarriers including endosome safety and effectiveness of long-term systemic use of ROS-responsive nanocarriers because of absence of degradability or insufficient biocompatibility. It seems that we need to pay more attention to develop clinically acceptable ROS-responsive nanocarriers with simpler and easier structure if we want to put these nanocarriers forward to commercialization as soon as possible. The safety and efficacy of ROS-responsive nanocarriers will be evaluated more precisely in biosystems. Moreover, the risks of ROS-responsive nanocarriers need to be considered for therapy application. The biological mechanisms underlying the interaction between active oxygen-based nanomedicines and the human body is not well-understood. The tissue structures and physiological behaviors of experimental animals, in which the drug carriers are often tested, are very different from humans. Moreover, different patients may react differently to these ROS-responsive nanomedicines because of the variations and complexities of the biological system. The specificity of ROS-responsive nanomedicines needs to improve to the specific patients with different tumors, inflammations, or neurodegenerative diseases in order to reduce security risk to normal tissues and organs. Hence, additional rigorous safety and effectiveness assessments of these nanocarriers should be carried out before they can be administered to patients. In conclusion, ROS-responsive nanocarriers have shown remarkable progress in preclinical studies over the past decade, and more clinical trials are needed to test their clinical utility.

## Author Contributions

TC and BC contributed to the design of the review. JL, YL, SC, and HL contributed to writing the paper. All authors approved the final version of the manuscript for submission.

## Conflict of Interest

The authors declare that the research was conducted in the absence of any commercial or financial relationships that could be construed as a potential conflict of interest.
